# PKCβ Phosphorylates PI3Kγ to Activate It and Release It from GPCR Control

**DOI:** 10.1371/journal.pbio.1001587

**Published:** 2013-06-25

**Authors:** Romy Walser, John E. Burke, Elena Gogvadze, Thomas Bohnacker, Xuxiao Zhang, Daniel Hess, Peter Küenzi, Michael Leitges, Emilio Hirsch, Roger L. Williams, Muriel Laffargue, Matthias P. Wymann

**Affiliations:** 1 Department of Biomedicine, University of Basel, Basel, Switzerland; 2 Medical Research Council, Laboratory of Molecular Biology, Cambridge, United Kingdom; 3 Friedrich Miescher Institute for Biomedical Research, Basel, Switzerland; 4 Biotechnology Centre, University of Oslo, Oslo, Norway; 5 Department of Genetics, Biology and Biochemistry, University of Torino, Torino, Italy; 6 INSERM, UMR1048, Institut des Maladies Métaboliques et Cardiovasculaires, Toulouse, France; The Babraham Institute, United Kingdom

## Abstract

All class I phosphoinositide 3-kinases (PI3Ks) associate tightly with regulatory subunits through interactions that have been thought to be constitutive. PI3Kγ is key to the regulation of immune cell responses activated by G protein-coupled receptors (GPCRs). Remarkably we find that PKCβ phosphorylates Ser582 in the helical domain of the PI3Kγ catalytic subunit p110γ in response to clustering of the high-affinity IgE receptor (FcεRI) and/or store-operated Ca^2+^- influx in mast cells. Phosphorylation of p110γ correlates with the release of the p84 PI3Kγ adapter subunit from the p84-p110γ complex. Ser582 phospho-mimicking mutants show increased p110γ activity and a reduced binding to the p84 adapter subunit. As functional p84-p110γ is key to GPCR-mediated p110γ signaling, this suggests that PKCβ-mediated p110γ phosphorylation disconnects PI3Kγ from its canonical inputs from trimeric G proteins, and enables p110γ to operate downstream of Ca^2+^ and PKCβ. Hydrogen deuterium exchange mass spectrometry shows that the p84 adaptor subunit interacts with the p110γ helical domain, and reveals an unexpected mechanism of PI3Kγ regulation. Our data show that the interaction of p110γ with its adapter subunit is vulnerable to phosphorylation, and outline a novel level of PI3K control.

## Abstract 2

The GPCR-activated PI3Kγ is also a key enzyme downstream of the IgE high affinity receptor FcεRI. PKCβ-dependent phosphorylation of PI3Kγ on Ser582 is the ‘missing link’ that functions as a molecular switch to divert PI3Kγ from GPCR inputs.

## Author Summary

Phosphoinositide 3-kinases (PI3Ks) are involved in most essential cellular processes. Class I PI3Ks are heterodimers: class IA PI3Ks are made up of one of a group of regulatory p85-like subunits and one p110α, p110β, or p110δ catalytic p110 subunit, and are activated via binding of their p85 subunit to phosphorylated tyrosine receptors or their substrates. The only, class IB PI3K member, PI3Kγ, operates downstream of G protein-coupled receptors (GPCRs). Recent work suggested that PI3Kγ also operates downstream of IgE-antigen complexes in mast cell activation, but no mechanism was provided. We show that clustering of the high-affinity IgE receptor FcεRI triggers a massive calcium ion influx, which leads to PKCβ activation. In turn, PKCβ phosphorylates Ser582 of the PI3Kγ catalytic p110γ subunit's helical domain. Downstream of GPCRs, p110γ requires a p84 adapter to be functional. Phospho-mimicking mutations at Ser582 disrupt the p84-p110γ interaction, and cellular Ser582 phosphorylation correlates with the loss of p84 from p110γ. Thus our data suggest that PKCβ phosphorylates and activates p110γ downstream of calcium ion influx, while simultaneously disconnecting the phosphorylated p110γ from GPCR signaling. Exploration of the p84-p110γ interaction surface by hydrogen- deuterium exchange mass spectrometry confirmed that the p110γ helical domain forms the main p84-p110γ contact surface. Taken together, the results suggest an unprecedented mechanism of PI3Kγ regulation.

## Introduction

Class I phosphoinositide 3-kinases (PI3Ks) produce the lipid second messenger phosphatidylinositol(3,4,5)-trisphosphate [PtdIns(3,4,5)*P*_3_] and consist of a p110 catalytic and a regulatory subunit. The class IA catalytic subunits, p110α, β, and δ, are constitutively bound to p85-related regulatory proteins that link them to the activation by protein tyrosine kinase receptors. The only class IB PI3K member, p110γ, is activated downstream of G protein-coupled receptors (GPCRs), and interacts with p101 or p84 (also known as p87^PIKAP^) adaptor subunits [1]–[3]. A tight complex of the p110γ catalytic subunit (PK3CG) with p101 was first discovered in neutrophils [1]. The p101 subunit (PI3R5) sensitizes PI3Kγ for activation by Gβγ subunits of trimeric G proteins, and is essential for chemotaxis of neutrophils towards GPCR-ligands [1],[4],[5].

Mast cells do not express p101; however, they do express the homologous adaptor protein p84 ([PI3R6]) [6], which shares 30% sequence identity with p101. Both p101 and p84 potentiate the activation of p110γ by Gβγ, but the p110γ-p101 complex is significantly more sensitive towards Gβγ, and displays an enhanced translocation to the plasma membrane as compared with p110γ-p84 [7]. Although p84 is absolutely required to relay GPCR signals to protein kinase B (PKB/Akt) phosphorylation and degranulation [6], its role is not completely understood: contrary to p110γ-p101, p110γ-p84 requires additionally the presence of the small G protein Ras, and might operate in distinct membrane micro-domains [6],[7].

Interestingly, genetic ablation of p110γ blocks high-affinity IgE receptor (FcεRI)-dependent mast cell degranulation in vitro and in vivo [8]. In part this is due to the fact that initial IgE/antigen-mediated mast cell stimulation triggers the release of adenosine and other GPCR ligands to feed an autocrine/paracrine activation of PI3Kγ, which then functions as an amplifier of mast cell degranulation. Interestingly, a substantial part of the observed PI3Kγ-dependent histamine-containing granule release (ca. 40%) was found to be resistant to *Bordetella pertussis* toxin (*P*Tx) pretreatment [9],[10]. Furthermore, although adenosine activates PI3Kγ via the A3 adenosine receptor (A_3_AR; [ADORA3]), A_3_AR null mice are still sensitive to passive systemic anaphylaxis, and degranulation in A_3_AR^−/−^ bone marrow-derived mast cells (BMMCs) upon antigen stimulation remains functional [11],[12]. This and the strong degranulation phenotype of PI3Kγ^−/−^ BMMCs suggest that GPCR signaling does not generate the full input to PI3Kγ-dependent degranulation, but a GPCR-independent activation mechanism for PI3Kγ has yet to be defined.

Here we identify a mechanism that activates PI3Kγ independently of GPCRs: we demonstrate that (i) IgE/antigen complexes and extracellular Ca^2+^ influx activate PI3Kγ, (ii) PI3Kγ is operationally linked to the FcεRI specifically by PKCβ (PRKCB), (iii) and that the phosphorylation of Ser582 located in the helical domain of p110γ by PKCβ leads to the dissociation of the p84 adapter to decouple phosphorylated p110γ from GPCR inputs. Further we characterize the p110γ-p84 interface, and delineate an activation process that seems to be conserved among class I PI3Ks.

## Results

### Thapsigargin-Induced Mast Cell Activation Needs PI3Kγ

**Figure 1:**
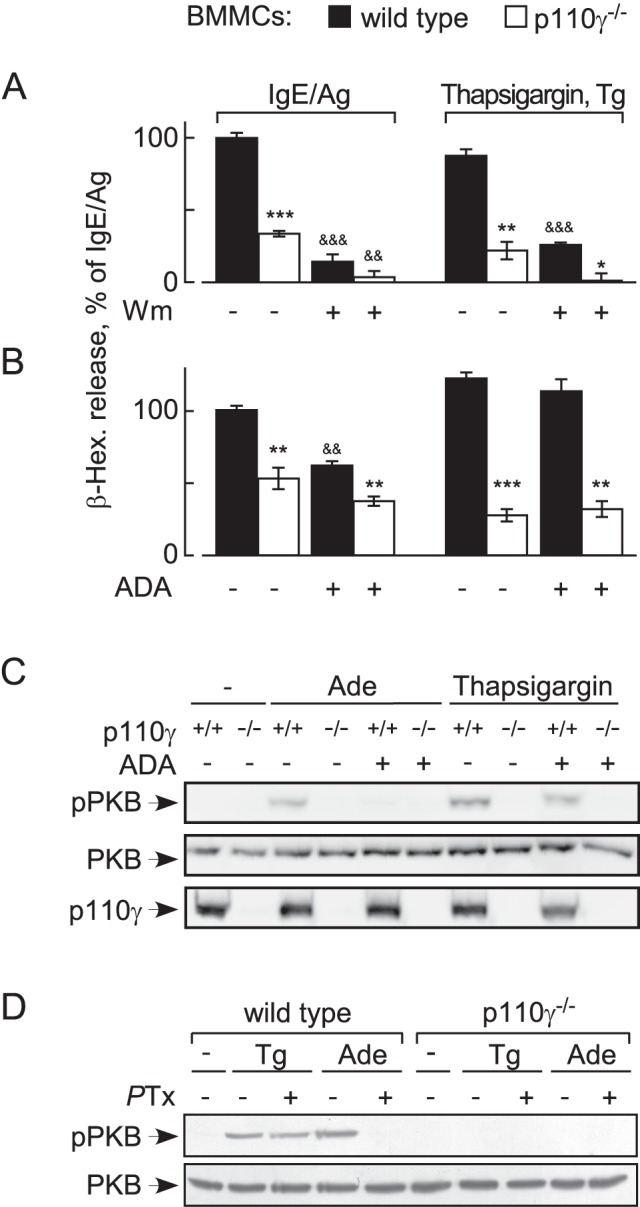
Thapsigargin-induced mast cell degranulation requires PI3Kγ, but not GPCR signaling. (A) Granule release of wild type and p110γ^−/−^ BMMCs was determined detecting β-hexosaminidase (β-Hex) release into extracellular media. BMMC stimulation with IgE/antigen was initiated with the antigen (Ag, DNP-HSA at 10 ng/ml; 100 ng/ml IgE overnight). Alternatively, BMMCs were stimulated by the addition of thapsigargin (1 µM). Where indicated, BMMCs were preincubated for 15 min with 100 nM wortmannin. Released β-Hex was quantified 20 min after stimulation, and is expressed as mean ± standard error of the mean (SEM) (*n* = 3; *p*-values in all figures are * or &: *p*<0.05, **: *p*<0.005; ***: *p*<0.0005; * depict here comparison with respective wild type control; & refer to comparison of untreated versus treated samples). (B) Granule release was assessed as above, but ADA (10 units/ml) was added to BMMC suspensions 1 min before stimulation where depicted. (C) Wild type or p110γ^−/−^ BMMCs were stimulated with adenosine (Ade; 1 µM) or thapsigargin (1 µM) for 2 min, and phosphorylation of PKB/Akt on Thr308 (pPKB), total PKB and p110γ was analyzed by Western blotting. BMMCs were incubated in starving medium (2% FCS, without IL-3) for 3 h before stimulation, and pretreated with ADA where indicated. (D) Heterotrimeric Gα_i_ proteins were inactivated by preincubation of wild type and p110γ^−/−^ BMMCs with 100 ng/ml *P*Tx for 4 h, before thapsigargin (Tg) or adenosine was added as in (C).

A committed step in mast cell activation is the influx of extracellular Ca^2+^ by store-operated Ca^2+^ entry (SOCE) [13]. Thapsigargin, which inhibits the sarco/endoplasmic reticulum Ca^2+^ reuptake ATPase (SERCA), causes depletion of Ca^2+^ stores, triggering SOCE. The latter achieves full-scale degranulation of BMMCs [14]. Surprisingly, BMMCs devoid of the p110γ catalytic subunit of PI3Kγ lost their responsiveness to thapsigargin and matched degranulation responses attained by wortmannin-pretreated cells (Figure 1A). To investigate if thapsigargin-triggered, p110γ-dependent degranulation involved release of adenosine, BMMCs were preincubated with adenosine deaminase (ADA) (Figure 1B) to convert adenosine to inosine, which has a very low affinity for adenosine receptors. ADA attenuated degranulation induced by IgE/antigen but did not affect thapsigargin-stimulated degranulation in wild type cells and did not further attenuate residual degranulation in p110γ null BMMCs. Likewise, presence of ADA did not reduce the phosphorylation of PKB/Akt in response to thapsigargin via the p110γ-dependent pathway (Figure 1C) but did reduce phosphorylation of PKB/Akt in response to adenosine—illustrating that the added ADA removes adenosine quantitatively. *P*Tx treatment of BMMCs (Figure 1D) blocked adenosine—but not thapsigargin-stimulated PKB/Akt phosphorylation.

To exclude that autocrine/paracrine signaling to p110γ occurred through *P*Tx-insensitive Gαq subunits to phospholipase β (PLCβ [PLCB2, PLCB3]), and a subsequent Ras activation by the Ras guanine nucleotide exchange factor RasGRP4 as described earlier in neutrophils [15], we used platelet activating factor (PAF) to trigger cyclic AMP-responsive element-binding protein (CREB) phosphorylation. PAF was reported earlier to trigger a *P*Tx-insensitive Ca^2+^ release from mast cells [9], and induced here a robust CREB phosphorylation comparable to adenosine and IgE/antigen. In contrast, PAF failed to trigger phosphorylation of PKB/Akt by itself, and did not enhance signaling of IgE/antigen to PKB/Akt (Figure S1).

Altogether, these results clearly illustrate that thapsigargin stimulates BMMCs via a PI3Kγ-dependent activation pathway, which operates separately from adenosine-induced activation of Gαi/o trimeric G proteins.

### Thapsigargin-Induced PI3Kγ Is Downstream of Extracellular Ca^2+^ Influx

**Figure 2:**
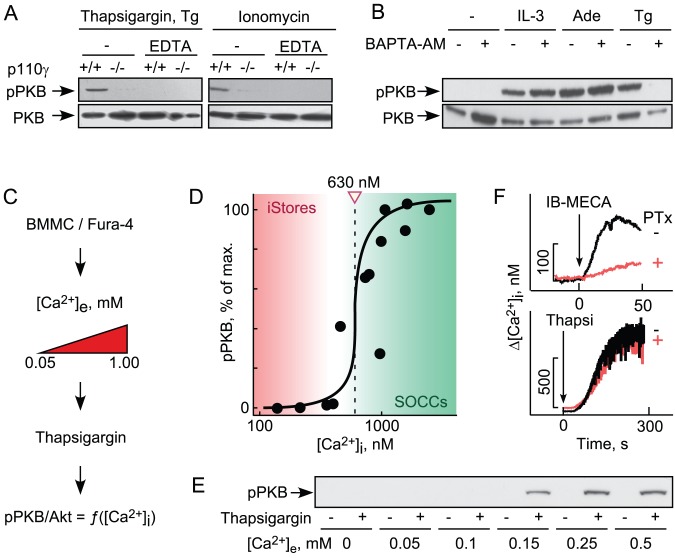
Thapsigargin-triggered PI3Kγ activation requires influx of extracellular Ca^2+^. (A) Where indicated, IL-3 starved BMMCs were incubated with EDTA (5 mM) for 5 min, before cells were stimulated with thapsigargin (1 µM) or ionomycin (1 µM). Cells were lysed 5 min after stimulation, and phosphorylation of PKB/Akt on Ser473 was analyzed. (B) BMMCs as in (A) were pretreated for 10 min with the cell-permeable Ca^2+^-chelator BAPTA/AM (10 µM) and stimulated either with IL-3 (10 ng/ml), adenosine (1 µM), or thapsigargin (1 µM). (C, D) BMMCs were loaded with the ratiometric low affinity Ca^2+^ probe Fura-4F/AM for 10 min in physiologic HEPES buffer at 1 mM Ca^2+^ (for details see Text S1). After the loading, washed cells were resuspended in the presence of increasing Ca^2+^ concentrations (extracellular Ca^2+^, [Ca^2+^]_e_) to modulate maximal stimulation-induced intracellular Ca^2+^ levels ([Ca^2+^]_i_). Cells were then stimulated with 0.5 µM thapsigargin, and maximal [Ca^2+^]_i_ increase and phosphorylation of PKB/Akt were measured. pPKB S473 levels are displayed as a function of the individually determined [Ca^2+^]_i_. Data points come from two independently performed experiments. (E) Representative anti-phospho-PKB/Akt immunoblot as used to determine pPKB/Akt levels in (D). (F) Intracellular Ca^2+^ concentrations were measured in wild type BMMCs following stimulation with the adenosine 3A receptor-selective agonist *N* ^6^-(3-iodobenzyl)-adenosine-5′-*N*-methylcarbox-amide (IB-MECA) (10 nM) or thapsigargin (1 µM). *B. Pertussis* toxin (100 ng/ml) was added 4 h before stimulation where marked.

PI3Kγ activation has been linked to ligation of GPCRs [1],[16], but not to elevated intracellular Ca^2+^ concentration ([Ca^2+^]_i_). We therefore chelated extracellular Ca^2+^ using EDTA, and buffered intracellular Ca^2+^ with the cell permeable Ca^2+^ chelator 1,2-Bis(2-aminophenoxy)ethane-N,N,N′,N′-tetraacetic acid tetrakis(acetoxymethyl ester) (BAPTA-AM). Extracellular and intracellular Ca^2+^ chelation prevented phosphorylation of PKB/Akt induced by thapsigargin or the Ca^2+^ ionophore ionomycin, while IL-3 and adenosine signaling to PKB/Akt remained unperturbed (Figure 2A and 2B).

Interestingly, the concentration of Ca^2+^ required to trigger PI3Kγ-dependent phosphorylation of PKB/Akt exceeded peak concentrations that are reached by GPCR stimulation: GPCR agonists release Ca^2+^ only from internal stores (maximum [Ca^2+^]_i_<300 nM), while thapsigargin (Figure 2C–2F) and IgE/antigen trigger SOCE and elevate [Ca^2+^]_i_ to µM concentrations [9],[14],[17]. Moreover, the correlation of maximally achieved [Ca^2+^]_i_ after thapsigargin revealed a steep, switch-like activation of PKB/Akt. While Ca^2+^ release from internal stores triggered by Gαi-coupled GPCRs (such as the A_3_AR stimulated by *N* ^6^-(3-iodobenzyl)-adenosine-5′-*N*-methylcarbox-amide [IB-MECA]) is sensitive to *P*Tx, thapsigargin-induced SOCE is not. Ca^2+^-induced activation of PI3Kγ only occurs after SOCE, and is therefore clearly separated from the GPCR/trimeric G protein/PI3Kγ axis.

### PKCβ Links Ca^2+^ Mobilization to PI3Kγ Activation

**Figure 3:**
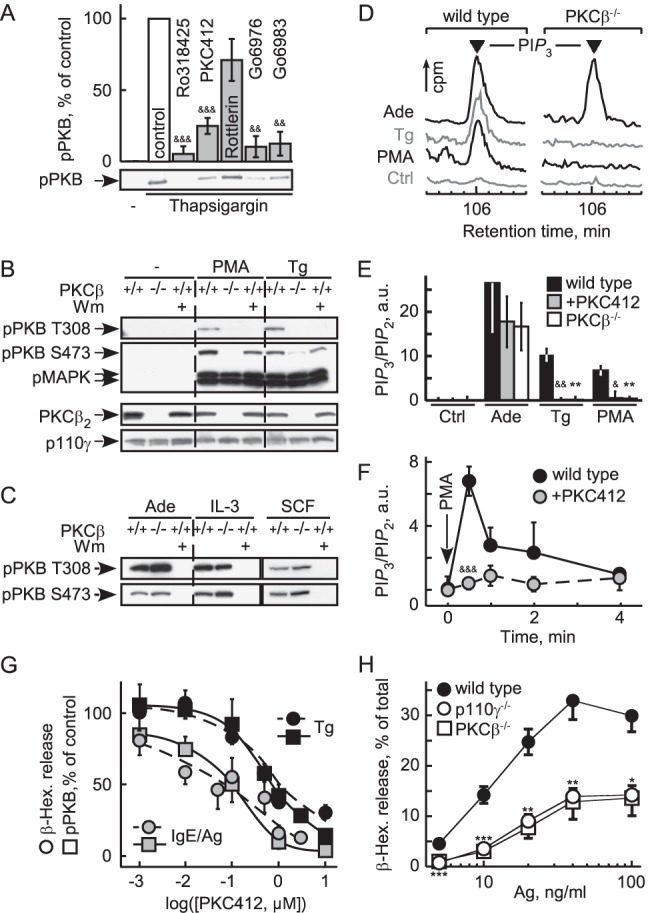
PKCβ relays thapsigargin-induced PI3Kγ activation. (A) Effect of PKC inhibitors on thapsigargin-induced PKB phosphorylation on Ser473 (S473). IL-3 starved BMMCs were preincubated with the indicated compounds for 20 min before stimulation (pan-PKC: Ro318425, Gö6983; classical PKC: PKC412 (CPG41251); classical and atypical PKC: Gö6976; Rotterlin: broad band inhibitor; see Text S1; & refers to comparison with untreated control; *p*-values see Figure 1). (B) PKB/Akt activation in response to 100 nM PMA or 1 µM thapsigargin was analyzed in wild type and PKCβ^−/−^ BMMCs. Cells were IL-3 deprived as in (A), and were pretreated with wortmannin (Wm, 100 nM) for 15 min before stimulation where indicated. Cells were lysed 2 min after stimulation, and analyzed for phosphorylation of PKB/Akt (T308 and S473) and MAPK (T183/Y185). (C) Wild type and PKCβ^−/−^ BMMCs were stimulated with 1 µM adenosine, 10 ng/ml IL3, or 10 ng/ml SCF, and processed as in (B). (D–F) PtdIns(3,4,5)*P*_3_ (PI*P*_3_) levels were determined in untreated (Ctrl) and classical PKC-inhibitor (PKC412)-treated wild type BMMCs and PKCβ^−/−^ BMMCs after stimulation with 0.5 µM thapsigargin, 200 ng/ml PMA, or 5 µM adenosine (30 s). BMMCs were metabolically labeled with [^32^P]-orthophosphate, lipids were extracted, deacylated, and applied to high-pressure liquid chromatography (HPLC). (D) shows representative elution peaks of PI*P*_3_ of the HPLC chromatograms. (E) Levels of PI*P*_3_ in relation to PtdIns(4,5)*P*_2_ (PI*P*_2_) were quantified by integration of the peak areas of PI*P*_3_ and PI*P*_2_ and expressed as ratio of PI*P*_3_/PI*P*_2_ (data shown as mean ± standard error of the mean [SEM], *n*≥4–6). (F) Cellular PI*P*_3_ production was measured over time in wild type BMMCs in response to PMA (200 nM) stimulation in the presence or absence of the classical PKC inhibitor PKC412 (mean ± SEM, *n* = 3). (G) Granule release and PKB activation (S473) in response to thapsigargin (1 µM) or IgE/antigen (100 ng/ml IgE overnight, 10 ng/ml DNP) was measured in the presence of increasing concentrations of the classical PKC inhibitor PKC412. Cells starved as in (A) were stimulated with IgE/antigen (IgE/Ag) or thapsigargin (Tg), and PKB phosphorylation and β-hexosaminidase release assays were performed in parallel (mean ± SEM, *n* = 3). (H) β-hexosaminidase release determined in wild type, PKCβ^−/−^, and p110γ^−/−^ BMMCs incubated with IgE, and stimulated with the indicated antigen (Ag) concentrations (mean ± SEM, *n* = 5; * refer to comparison with wild type control. Only the higher *p*-values of the overlapping data points are indicated).

Protein kinase C (PKC) inhibitors (Ro318425, Gö6983, Gö6976) targeting classical and atypical PKCs, and the inhibitor PKC412, which mainly inhibits classical PKCs, all substantially blocked PKB/Akt phosphorylation in response to thapsigargin and phorbol 12-myristate 13-acetate (PMA) (Figures 3A and S2A). Rottlerin, with a limited selectivity for PKCδ, had no effect on PKB/Akt activation. GPCR-dependent PI3Kγ activation by adenosine was resistant to all tested PKC inhibitors (Figure S2B). The inhibitor profile suggested that a classical PKC activates PI3Kγ. While PKB/Akt activation by PMA and thapsigargin was blocked in PKCβ^−/−^ BMMCs (Figure 3B), signaling in PKCα^−/−^ and PKCγ^−/−^ BMMCs remained intact (Figure S2C). Deletion of PKCβ eliminated phosphorylation of PKB/Akt on Thr308 and Ser473 completely, whereas a residual signal on Ser473 was observed after PI3K-inhibition by wortmannin. This may be explained by the observation that PKCβ2 can function as a Ser473 kinase [18]. Adenosine, IL-3, and stem cell factor (SCF)-induced PKB/Akt activation was not affected by elimination of PKCβ (Figure 3C), demonstrating that PKCβ does not relay adenosine signals to PI3Kγ, and is not required in cytokine and growth factor receptor-dependent activation of class IA PI3Ks in mast cells.

The direct measurement of phosphoinositides in BMMCs confirmed that ablation of PKCβ or its inhibition eliminated production of PtdIns(3,4,5)*P*_3_ triggered by thapsigargin and PMA, but not adenosine (Figure 3D–3F). Interestingly, the link between PKCβ and PI3Kγ seems to be transient in nature, as PMA stimulation triggers short lived PtdIns(3,4,5)*P*_3_ peaks (Figure 3F). Impaired FcεRI-triggered degranulation has been reported in both p110γ^−/−^ [9] and PKCβ^−/−^ BMMCs [19], and the sensitivity of degranulation to PKC inhibition fits the phospho-PKB/Akt output (Figure 3G). This, combined with the similarity of p110γ and PKCβ null phenotypes in IgE/antigen-induced degranulation (Figure 3H), suggests a direct link of PKCβ and PI3Kγ downstream of FcεRI.

### PKCβ Binds and Phosphorylates PI3Kγ

**Figure 4:**
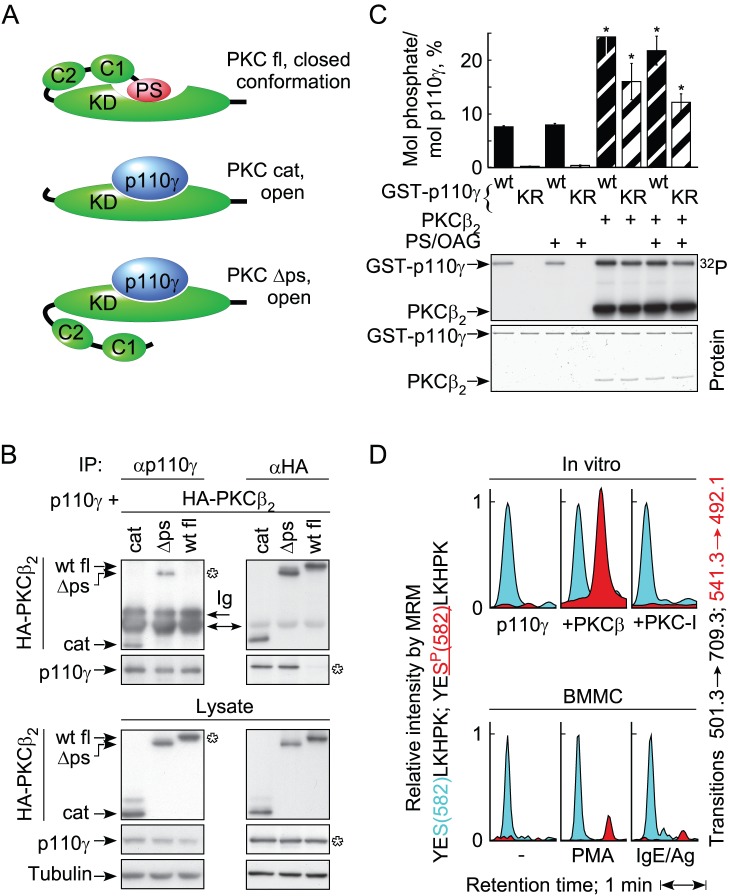
PKCβ interacts with and phosphorylates the catalytic subunit of PI3Kγ. (A) Schematic representation of the PKCβ-p110γ interaction: full-length (fl) PKCβ is in a closed conformation due to the interaction of the pseudo-substrate domain with the catalytic pocket of PKCβ, while the truncated catalytic domain (cat; amino acids 302–673) and pseudo-substrate deletion mutant (Δps; deletion of aa 19–31) give access to p110γ. (B) HEK293 cells were co-transfected with p110γ and HA-tagged PKCβ2 constructs. Protein complexes were immunoprecipitated with anti-p110γ or anti-HA antibodies, before HA-PKCβ2 and p110γ was detected by immunoblotting. Ig: immunoglobulin heavy chain signals of mouse anti-p110γ and anti-HA antibodies. (C) Recombinant GST-p110γ wild type (wt) or a catalytically inactive p110γ mutant (KR, Lys833Arg mutant) were incubated with recombinant PKCβ2 and [γ^32^P]-ATP in kinase buffer for 30 min, before proteins were denatured and separated by SDS-PAGE. Phosphatidylserine (PS) lipid vesicles containing 1-oleoyl-2-acetyl-sn-glycerol (OAG) were present during the reaction where marked. Protein-bound ^32^P was determined by radioisotope imaging, and recombinant proteins were stained with Coomassie blue (mean ± standard error of the mean [SEM], *n* = 3; * point to comparison with respective sample without PKC). (D) In vitro and in vivo phosphorylation of PI3Kγ on S582, analyzed by LC-MRM. S582 non-phospho- and phospho-peptides were detected in the MRM mode, quantifying the transition 501.1 to 709.3 for the non-modified peptide (blue) and 541.3 to 492.1 for the phospho-peptide (red). Data were normalized to the transition of the non-modified peptide, which was set to 1. Upper part: recombinant catalytically inactive human GST-PI3Kγ (2 µg) was incubated alone, together with PKCβ2 or with PKCβ2 and PKC-inhibitor (Ro318425, 2 µM) as in (C). After SDS-PAGE and Coomassie staining, PI3Kγ was excised from the gel and prepared for LC-MRM. Lower part: wild type BMMCs (300 M cells/stimulation) were starved for 4 h in IL-3 free medium/2% FCS, and were left unstimulated or were treated for 2 min with 50 nM PMA or for 4 min with 10 ng/ml antigen (cells preloaded with 100 ng/ml IgE overnight). Endogenous PI3Kγ was immunoprecipitated from cell lysates, resolved by SDS-PAGE and analyzed with LC-MRM.

Co-expression of p110γ with tagged full length or truncated PKCβ2 (Figure 4A) revealed that only the catalytic domain fragment and a pseudo-substrate deletion mutant of PKCβ2 formed complexes with p110γ (Figure 4B), suggesting that the presence of the pseudo-substrate in PKCβ results in a closed conformation that is unable to interact with p110γ. An in vitro protein kinase assay with recombinant PKCβ2 and glutathione S-transferase (GST)-tagged wild type p110γ or catalytically inactive p110γ (KR; Lys833Arg mutant) as substrate, showed that PKCβ robustly phosphorylated p110γ (Figure 4C). The capability of p110γ to auto-phosphorylate [20] was not required in the process.

Analysis of phosphorylated, catalytically inactive p110γ by liquid chromatography tandem mass spectrometry (LC-MS/MS) identified Ser582 as a target residue of PKCβ (YES^P^[582]LKHPK; spectra in Figure S3). Mass spectrometric multiple reaction monitoring (MRM) (Figure S3C) showed that Ser582 phosphorylation was absent in assays lacking PKCβ, or when PKC-inhibitor was added (Figure 4D). Ser582 phosphorylation was also detected by MRM in PMA and IgE/antigen stimulated BMMCs (Figure 4D, lower panel).

**Figure 5:**
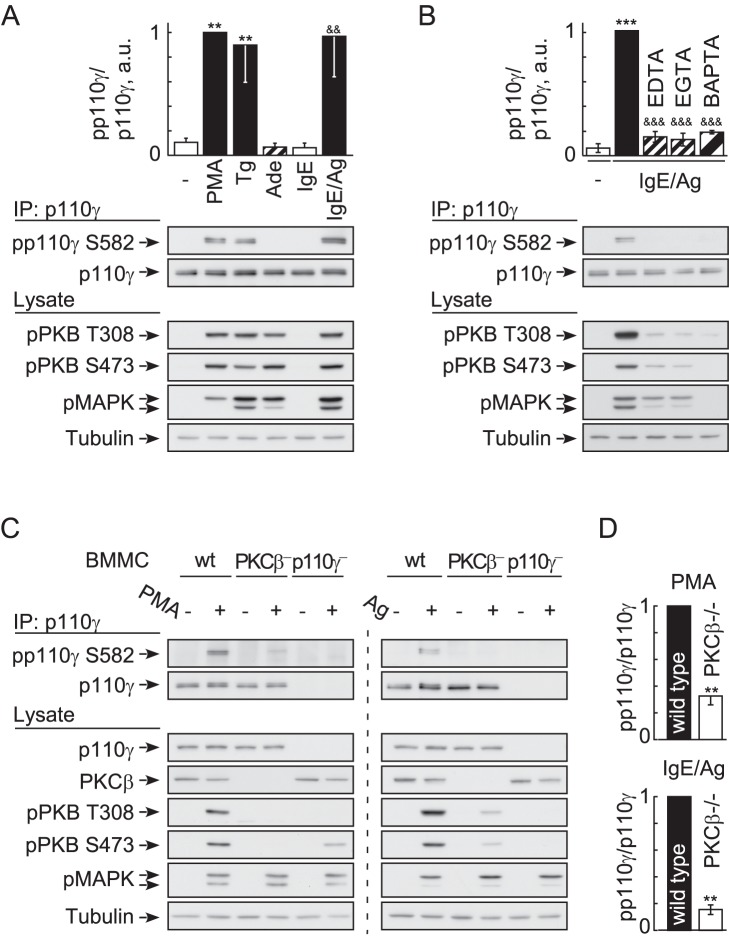
Phosphorylation of PI3Kγ requires Ca^2+^ and is PKCβ-dependent. (A) Stimulus-induced phosphorylation of endogenous p110γ on Ser582 in wild type BMMCs. IL-3 deprived cells were stimulated with 100 nM PMA, 1 µM thapsigargin, 1 µM adenosine, or 20 ng/ml DNP for 2 min. Where indicated (IgE), BMMCs were loaded with IgE (100 ng/ml) overnight. PI3Kγ was immunoprecipitated from cell lysates with an anti-PI3Kγ antibody, before precipitated protein was probed for phosphorylated p110γ (pp110γ) with a phospho-specific anti-pSer582 antibody (validation of antibody see Figure S4). PI3Kγ phosphorylation is shown normalized to total PI3Kγ levels (mean ± standard error of the mean [SEM], *n* = 3; * depict analysis using unstimulated control. & reference point is IgE only). (B) IgE/antigen-induced Ser582 phosphorylation of p110γ requires Ca^2+^ influx. Cells were stimulated as in (A), but exposed to EDTA, EGTA, or loaded with BAPTA/AM where indicated (see Figure 2). Phosphorylated p110γ was detected as in (A); mean ± SEM, *n* = 3; * comparison with unstimulated control; ^&^analysis of stimulated versus chelator treated). (C) Phosphorylation of p110γ in wild type and PKCβ^−/−^ BMMCs. Experimental settings were as in (A), and (D) depicts quantification of pp110γ in relation to total p110γ protein (mean ± SEM; PMA *n* = 4, antigen *n* = 3). Cells devoid of p110γ were included as negative control.

Phospho-Ser582 site-specific antibodies (see Figure S4) revealed p110γ Ser852 phosphorylation in PMA-, thapsigargin-, and IgE/antigen-stimulated BMMCs, but not in mast cells exposed to adenosine or IgE alone (Figure 5A). Consistent with the requirement of Ca^2+^ mobilization, IgE/DNP-induced phosphorylation of p110γ on Ser582 was blocked by extracellular (EDTA, EGTA) and intracellular (BAPTA/AM) Ca^2+^ chelation (Figure 5B).

Although the extended peptide around Ser582 scores as a PKC substrate site, the core Arg-X-X-Ser582 sequence is a putative recognition site for several protein kinases (scores are PKC>protein kinase A>calcium/calmodulin-dependent kinases [CAMK]). As mast cell activation is accompanied by a massive influx of extracellular Ca^2+^, we assessed if CAMK could phosphorylate p110γ directly. In the presence of ^32^P-γ-ATP, recombinant CAMKII (CAMK2) incorporated equal amounts of phosphate into free and p84-bound p110γ. In the same experiment, PKCβ preferentially phosphorylated free p110γ, illustrating a preference of PKCβ for p110γ surfaces obscured in the p84-p110γ complex. CAMK also substantially phosphorylated p84, which was borderline in in vitro assays with PKC (12 versus 3 mol %) (Figure S5A). Probing the phosphorylation of Ser582 in vitro demonstrated that access to this site is blocked when p84 is bound to p110γ (Figure S5B).

In cellular assays stimulating BMMCs with thapsigargin, CAMK activation could be monitored using anti-phospho-CAMKII antibodies. While PKC inhibitors left CAMKII phosphorylation >50% intact, Ser582 and PKB/Akt phosphorylation were both reduced to background levels (Figure S6), even in a context favoring Ca^2+^-triggered responses. The above, and the fact that PKCβ^−/−^ BMMCs showed a major reduction in phospho-Ser582 after PMA or IgE/antigen stimulation (Figure 5C and 5D), illustrate that phosphorylation of p110γ is mainly mediated by PKCβ, and that other PKC isoforms (see also Figure S2) and Ca^2+^-dependent kinases attribute to less than 18% of the observed overall signal.

### Phosphorylation of Ser582 Positively Regulates p110γ's Activity and Displaces p84

**Figure 6:**
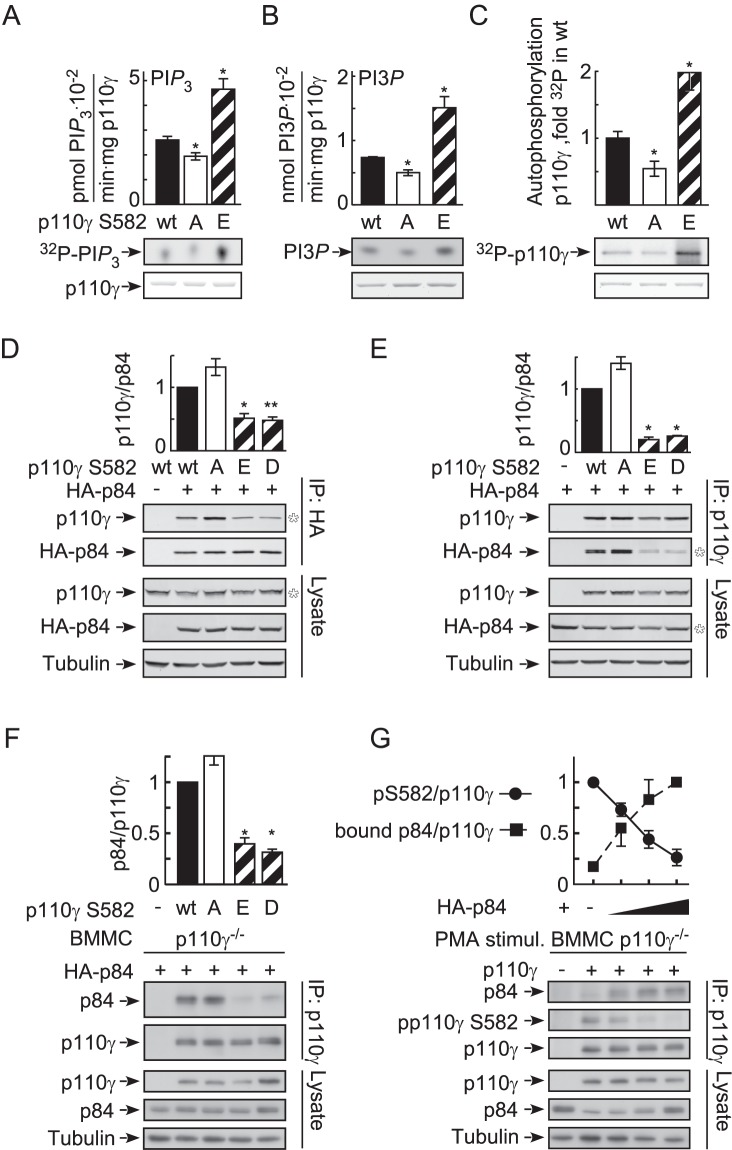
Ser582 phosphorylation increases p110γ activity and displaces p84. (A–C) Lipid and protein kinase activities of recombinant p110γ wild type (wt) and phosphorylation site mutants (Ser582Ala, [A]; Ser582Glu, [E]). (A) Mixed phospholipid vesicles containing PtdIns(4,5)*P*_2_ were used to measure PtdIns(3,4,5)*P*_3_ (PI*P*_3_) production, while in (B) PtdIns was used as a substrate. Protein kinase activity of p110γ was determined by auto-phosphorylation in (C). Bars display quantifications of incorporated ^32^P as mean ± standard error of the mean [SEM] (*n* = 3; * comparison with wt protein), and representative thin layer chromatograms [^32^P] and loaded p110γ protein (Coomassie blue) are shown below. (D, E) Wild type p110γ and Ser582 mutants (Ser582Asp, [D]) were expressed together with HA-tagged p84 in HEK293 cells. In (D), the PI3Kγ complex was immunoprecipitated with anti-HA antibodies directed against HA-p84 (*n* = 4). In (E) PI3Kγ was immunoprecipitated using anti-p110γ antibodies (*n* = 2). (F) Wild type p110γ and Ser582 mutants were co-expressed with p84 in p110γ^−/−^ BMMCs. PI3Kγ was immunoprecipitated from cell lysates as in E (mean ± SEM, *n* = 3). (G) The p84 adapter competes with PKCβ for access to p110γ. Increasing amounts of HA-p84 expression plasmid were transfected into p110γ^−/−^ BMMCs, before the cells were stimulated with PMA for 30 s. PI3Kγ was subsequently immunoprecipitated with an anti-p110γ antibody, and bound p84 and Ser582 phosphorylation were quantified. The values are normalized to total p110γ protein (mean ± SEM, *n* = 2).

To evaluate if the Ser582 phosphorylation affected the intrinsic activity of p110γ, a phosphorylation-mimicking mutant (Ser582Glu) was produced. The activity of the p110γ Ser582Glu mutant was enhanced approximately 2-fold independently of the substrate used (PtdIns(4,5)*P*_2_, PtdIns, or auto-phosphorylation) (Figure 6A–6C). Ser582 is localized in the helical domain of p110γ, and it is interesting to note that helical domain mutants of p110α found in tumors display a similar increase in enzyme activity [21]–[25].

As mutations in the helical domain of p110α attenuate contacts with the p85 regulatory subunit [21], we examined binding of p110γ mutants to the PI3Kγ adaptor subunit p84: the substitution of Ser582 with Glu and Asp, abrogated p110γ-p84 interactions in HEK293 cells and BMMCs, while the Ser582Ala replacement favored p110γ-p84 complex formation (Figure 6D–6F). In line with this, PMA-induced phosphorylation of Ser582 in BMMCs was suppressed by the overexpression of p84 (Figure 6G), which fits the very limited access of PKCβII to in vitro phosphorylate Ser582 in the p110γ-p84 complex (reduced to 20% of phosphorylation of free p110γ) (Figure S5B).

Most importantly, the correlation of phosphorylation of Ser582 on p110γ and the release of p84 could also be established in wild type BMMCs: when stimulated with PMA, the amount of p84 that could be co-precipitated with p110γ was reduced significantly, and was linked to Ser582 phosphorylation of p110γ. In the inverse co-immunoprecipitation, anti-p84-associated p110γ was reduced, and phosphorylation was below detection levels in the remaining p84-associated p110γ (Figure S7). The collected results are in agreement with a mechanism in which PKCβII-mediated phosphorylation of Ser582 and the interaction of p84 and p110γ are exclusive events, and in which PKCβ action displaces p84 from p110γ.

### The Helical Domain of p110γ Binds and Is Stabilized by p84

In order to understand how p84 could mask Ser582 phosphorylation, and to map the p110γ-p84 contact interface, hydrogen deuterium exchange mass spectrometry (HDX-MS) was used. HDX-MS elucidated contacts of class IA p110δ with its p85 regulatory subunit, and the mechanism of action of cancer-linked mutations in p110α [25],[26]. HDX-MS relies on amide hydrogen exchange with solvent at a rate dependent on their involvement in secondary structure and solvent accessibility. Following proteolysis, location and extent of deuterium uptake are analyzed by peptide mass determination. The primary sequence of p110γ was covered >90% by 202 peptide fragments (Figure S8; Table S1).

**Figure 7:**
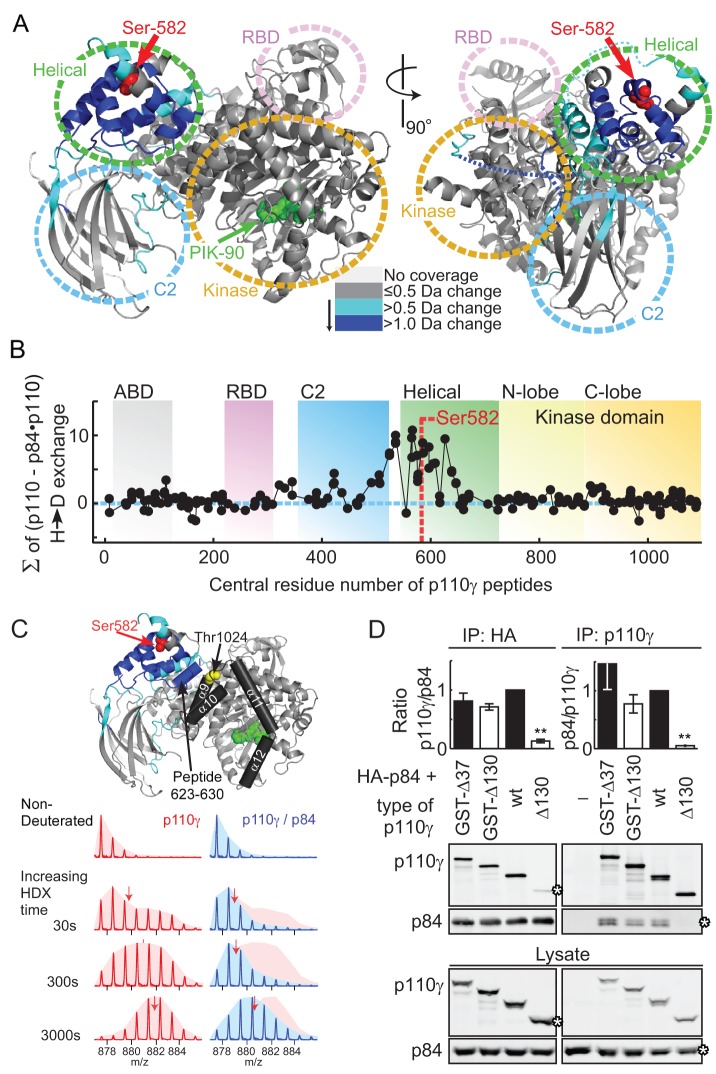
p84 Interacts with the helical domain of p110γ. (A) Changes in deuteration levels between free and p84-bound PI3Kγ are mapped onto the crystal structure of PI3Kγ (PDB ID: 2CHX). Regions that are covered by peptides of PI3Kγ (labeled A–R) that showed greater than 0.5 or 1.0 Da changes in deuteration are colored light or dark blue, respectively. The greatest difference in exchange observed at any time was used for the mapping. S582 is labeled red. The ATP competitive inhibitor PIK-90 in the crystal structure is shown in green as a reference point for the kinase domain. The linker regions between the RBD and the C2 domain and the C2 and the helical domain are shown as dotted lines (right part). (B) The percent deuterium exchange differences between free and p84-bound PI3Kγ were summed up over all seven time points for every identified peptide (*y*-axis), which were graphed according to their central residue number (*x*-axis). (C) A selected peptide (623–630) from the helical domain is shown at four time points of H/D on-exchange +/− the p84 subunit. In the absence of the p84 adaptor the majority of peptides in the helical domain showed broadening of the isotopic profiles indicative of EX1 kinetics (see 30, or 300 s in free p110γ). The helix A3 (624–631) selected is located at the interface of the helical domain with the C-lobe. Ser582 and Thr1024 have been highlighted as a reference. (D) p84 was coexpressed with GST-tagged or untagged PI3Kγ constructs in HEK293 cells. N-terminal deletions of 37 or 130 amino acids are denoted Δ37 or Δ130, respectively. HA-p84 (left) or PI3Kγ (right) was immunoprecipitated from cell lysates with anti-HA or anti-PI3Kγ antibodies and protein G beads. PI3Kγ-p84 interactions were analyzed by Western blotting, quantified with Odyssey Imager software and expressed as fold of untagged, full-length p110γ-p84 association (mean ± standard error of the mean [SEM], left: *n* = 4, 6, 6, 6; right: *n* = 2, 4, 4, 4).

Deuterium (^2^H) incorporation into free and p84-complexed p110γ was analyzed at seven time points (3 to 3,000 s). Differences in ^2^H-exchange of free and complexed p110γ were mapped onto the crystal structure of p110γ lacking the N-terminal domain (PDB ID:2CHX, residues 144–1,093), to visualize conformational changes induced by p84 (Figures 7A, S9, and S10). Peptides with highest decrease in ^2^H incorporation (>1.0 Da) clustered to the RBD-C2 linker, the C2-helical domain linker, and the helical domain. The ^2^H-incorporation in the presence of p84 is visualized as integrated average difference in exchange at all seven time points in Figure 7B, illustrating that the helical domain provides the dominant interface with p84. Due to difficulties in producing free p84, the contacts on p84 with p110γ could not be mapped. Interestingly, in the absence of the p84 subunit, the majority of peptides in the helical domain exhibited broad isotopic profiles (HDX of peptide 623–630, which is representative of peptides in the helical domain, is shown in Figures 7C and S11). This type of profile known as type 1 exchange (EX1) kinetics is indicative of concerted dynamic motions of a substructure in a protein, rather than the local fluctuations characteristic of EX2 kinetics [27].

The N-terminus of p110γ was shown to stabilize the p110γ-p101 heterodimer [28]. Expression of p110γ mutants lacking the first 130 amino acids (Δ130–p110γ) seemed to support this view, as association with p84 was lost (Figure 7D). However, when truncated p110γ was N-terminally tagged with GST (GST-Δ130-p110γ), binding of p84 was restored. Although we detected a small decrease in the ^2^H-incorporation in two N-terminal p110γ peptides (59–70 and 107–113) in the presence of p84, this interaction seems to be dispensable. The N-terminus of p110γ instead has a role in stabilizing the intact catalytic subunit. The helical domain is the main location of interaction with p84; however, it appears that this interaction is vulnerable and easily broken, as a single phosphorylation at Ser582 is able to disrupt the contact.

## Discussion

The activation of PI3Kγ has been tightly linked to GPCRs-triggered dissociation of trimeric Gαi proteins, and has been shown to require the interaction of Gβγ subunits with p110γ and the PI3Kγ adaptor subunits p101 and p84 [1],[2],[4],[16],[29],[30]. Moreover, GPCRs generate PI3K signals typically through PI3Kγ, thereby controlling extravasation of hematopoietic cells [31],[32], cardiovascular parameters [33],[34], and metabolic output [35],[36].

Non-GPCR-mediated activation of PI3Kγ has not been reported so far, but it has been shown that phorbol esters and Ca^2+^ ionophores can modulate phosphoinositide levels in a variety of cells, including platelets [37], adipocites [38], fibroblasts [39], and hematopoietic cells [40]. The proposed mechanisms have been diverse and involved protein tyrosine kinases and GPCR signaling. A recent finding that protein kinase D (PKD) can phosphorylate two distinct sites on the p85 regulatory subunit to control class IA PI3K activity [41] is an indication that PI3K control is more complex than anticipated.

PI3Kγ has been shown to be a key element in enhancing IgE/antigen output by the release of adenosine. This process involves signaling downstream of Gαi-coupled A_3_AR, and is sensitive to *P*Tx and ADA [9]. The resistance of thapsigargin-induced degranulation to ADA shown here, and the fact that *P*Tx did not diminish the PI3Kγ-dependent, thapsigargin-induced phosphorylation of PKB/Akt, points to a novel mechanism of PI3Kγ activation, which is clearly distinct from GPCR action. This Ca^2+^-mediated PI3Kγ activation requires SOCE and [Ca^2+^]_i_ >600 nM. In contrast, GPCRs yield phosphorylation of PKB/Akt in mast cells even in the absence of a change in [Ca^2+^]_i_. Furthermore, increases in [Ca^2+^]_i_ triggered via GPCRs remain at levels incapable of engaging a Ca^2+^-dependent activation of PI3Kγ.

Thapsigargin bypasses the signaling chain from IgE/antigen-clustered FcεRI to the activation of phospholipase Cγ (PLCγ) and inositol(1,4,5)-trisphosphate (Ins(1,4,5)*P*_3_) production, and triggers SOCE by the depletion of Ca^2+^ stores. That thapsigargin requires functional PI3K activity to induce mast cell degranulation was first demonstrated using the PI3K inhibitors wortmannin and LY294002 [14], but no link between [Ca^2+^]_i_ rise and PI3Kγ activity was established previously. The fact that inhibitors for classical PKCs only prevented the thapsigargin- and PMA-induced phosphorylation of PKB/Akt, while the GPCR-mediated phosphorylation of PKB/Akt remained intact, points to a link between classical PKCs and PI3Kγ. Experiments on BMMCs lacking PKCα, PKCβ, and PKCγ, showed that only the PKCβ null cells lost the ability to activate PKB/Akt in response to thapsigargin or PMA stimulation. As the PMA- and thapsigargin-induced phosphorylation of PKB/Akt on Ser473 showed a partial resistance to wortmannin, and as it has been reported that PKCβ can directly phosphorylate Ser473 in the hydrophobic motif of PKB/Akt [18], the effect of genetic and pharmacological targeting of PKCβ was also validated measuring PtdIns(3,4,5)*P*_3_ production directly. The lack of PtdIns(3,4,5)*P*_3_ production in thapsigargin or PMA-stimulated BMMCs treated with the PKC inhibitor PKC412, and in cells devoid of PKCβ, is in agreement with a requirement of PKCβ upstream of PI3Kγ. That signaling from PKC to PI3K plays a role in mast cell degranulation is further supported by the close correlation of PKC inhibitor sensitivity of phosphorylated PKB/Akt and degranulation responses. Moreover, the loss of PKCβ or PI3Kγ results in a similar reduction of degranulation over a wide range of IgE/antigen concentrations. The results obtained here are in agreement with previous findings that mast cells and mice devoid of p85α/p55α/p50α [42],[43] and p85β [44] remain fully responsive to IgE/antigen complexes. A previous report showed a biphasic activation of PI3K with PI3Kγ having an early role and PI3Kδ a later role downstream of FcεRI in murine mast cells [45],[46].

A mechanistic link between PKCβ and the catalytic subunit of PI3Kγ was initially difficult to establish, as the direct PtdIns(3,4,5)*P*_3_ response to PMA-stimulation was transient (main peak half life <1 min), and the two full-length enzymes interacted only weakly. The observation that truncated, activated forms of PKCβ formed stable complexes with p110γ, suggested that PKCβ must attain an open conformation to interact with p110γ, and that PKCβ binds to p110γ via its catalytic domain. This contact resulted in phosphorylation of Ser582 on p110γ, which could be detected both in vitro and in PMA or IgE/antigen-stimulated BMMCs by mass spectrometry. The PKCβ-mediated phosphorylation of p110γ was confirmed using site-specific anti-phospho-Ser582 antibodies. Stimuli like PMA, thapsigargin, and IgE/antigen complexes all required PKC to signal to PKB/Akt, which correlated with the phosphorylation of Ser582 on p110γ. Moreover, the phosphorylation of Ser582 on p110γ was sensitive to removal of extracellular Ca^2+^, buffering of [Ca^2+^]_i_, and the genetic deletion of PKCβ. Adenosine stimulates PI3Kγ via GPCRs and *P*Tx-sensitive trimeric G proteins [9] in a Ca^2+^-independent process, and did not yield a detectable phosphorylation of Ser582.

The increased turnover of PtdIns and PtdIns(4,5)*P*_2_, and the increased rate of auto-phosphorylation displayed by p110γ with a phosphate-mimicking mutation (Ser582Glu), suggests that a structural change in the helical domain of p110γ is sufficient to increase the catalytic activity independent of the presence of the p84 subunit. Previous work examining the activation of the class IA p110α, p110β, and p110δ catalytic subunits has shown that part of the activation mechanism occurs through a conformational change from a closed cytosolic form to an open form on membranes [25],[26]. The helical domain of p110γ is exquisitely well placed to propagate conformational changes due to the fact that it is in contact with every other domain in p110γ. In the crystal structure of N-terminally truncated (Δ144) p110γ, the side chain of Ser582 points inward [47],[48], and has to rotate to accommodate a phosphate. Our HDX-MS results showed a dynamic “breathing” motion in the helical domain in the free p110γ catalytic subunit that may allow for temporary exposure of Ser582, enabling modification by PKC. HDX results showed that the p84 subunit slowed or prevented this dynamic motion, and this correlated with a decreased efficiency of phosphorylation by PKC in cells in the presence of p84.

Although Ser582 is not in a direct contact with the kinase domain, it is structurally linked to it: the heat repeat HA1/HB1 housing Ser582, and the connecting intra-helical loop (residues 560 to 570), along with helix A3 (624–631) are in contact with helices kα9 and kα10 in the C-lobe of the kinase domain (known as the regulatory arch [49],[50]) and could transduce a conformational change to the catalytic center of p110γ (Figure 7C). The phosphorylated Ser582 and phosphorylation-mimicking mutants may activate lipid kinase activity by causing a conformational shift at this interface. It has been shown recently that this region of the p110γ kinase domain is critical in regulating lipid kinase activity, as phosphorylation of Thr1024 in the kα9 by protein kinase A (PKA) negatively regulates p110γ activity in vitro and in cardiomyocytes (Figure 7C) [51].

**Figure 8:**
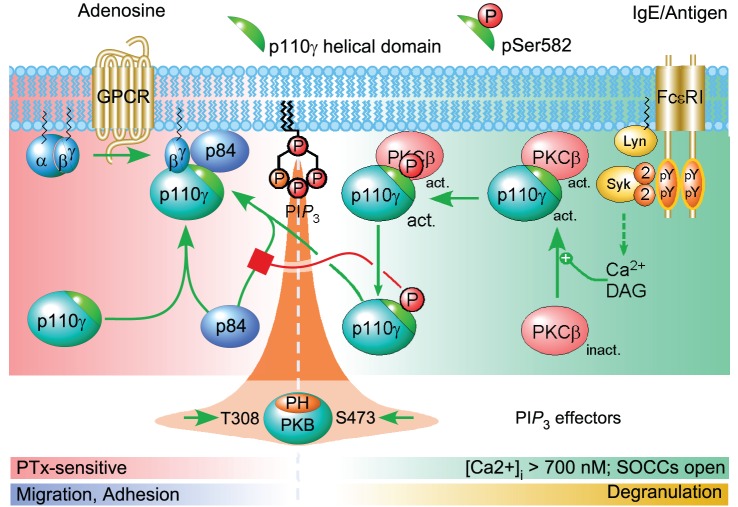
Phosphorylation of Ser582—loss of GPCR coupling of p110γ. In a resting mast cell, the PI3Kγ complex is responsive to GPCR-mediated dissociation of trimeric G proteins. An adapter protein (here p84) is required for a productive relay of the GPCR signal to PI3Kγ. When FcεRI receptors are clustered via IgE/antigen complexes, a signaling cascade is initiated, which triggers the depletion of intracellular Ca^2+^ stores and the opening of store-operated Ca^2+^ channels. The resulting increase in [Ca^2+^]_i_ and PLCγ-derived diacylglycerol activate PKCβ, which binds to p110γ and subsequently phosphorylates Ser582 (→pp110γ). Phosphorylated p110γ cannot interact with p84, and is therefore unresponsive to GPCR inputs. GPCR input to PI3Kγ coincides with migration and adhesion, while Ca^2+^/PKCβ activation of p110γ occurs when mast cells degranulate. The phosphorylation of PKB/Akt occurs downstream of PtdIns(3,4,5)*P*_3_, which originates from G protein-activated p84-p110γ complex or PKCβ-activated pp110γ. The phosphorylated residues Thr308 and Ser473 of PKB/Akt are used to monitor PI3K activation. More detailed effector signaling event schemes can be found in [52].

In contrast to the p110α-p85 heterodimer, stabilized by the N-terminal adaptor-binding domain (ABD)/inter SH2 domain interaction, the association of the p110γ subunit with its adaptor subunit is quite vulnerable, and the Ser582Glu mutant, but not Ser582Ala, abrogated the formation of a p110γ-p84 complex. HDX-MS revealed that the helical domain of p110γ was stabilized by p84. Ser582 is located in the center of the p110γ-p84 contact surface, which explains how a change in charge (Ser582Glu) breaks the interaction with p84, either by direct contact or by destabilization of the helical domain. Overexpression of p84 shields p110γ from a PMA-induced phosphorylation, and suggests that binding of p84 to p110γ, and Ser582 phosphorylation by PKCβ are mutually exclusive. This implies that the two activation modes of PI3Kγ—by GPCRs or PKCβ—are completely separated. At low [Ca^2+^]_i_, PI3Kγ is exclusively activated by Gβγ subunits. It has been demonstrated that a PI3Kγ adapter protein is absolutely needed for functional GPCR inputs to p110γ [6]. If the interaction of p84 with p110γ is blocked by the phosphorylation of Ser582, p110γ is decoupled from its GPCR input (for a schematic view of the process see Figure 8). The PKCβ-mediated activation and phosphorylation of p110γ constitutes therefore an unprecedented PI3K molecular switch, which enables the operation of p110γ downstream of FcεRI signaling, and will elucidate cell type-specific activation processes in allergy and chronic inflammation.

## Materials and Methods

### Cells and Mice

BMMCs were derived from bone marrow of 8–12-wk-old C57BL/6J wild type, p110γ^−/−^, PKCα^−/−^, PKCβ^−/−^, and PKCγ^−/−^ mice, and cultured and characterized as described in [9]. Animal experiments were carried out in accordance with institutional guidelines and national legislation. Human embryonic kidney 293 (*HEK293*) cells were grown in DMEM supplemented with 10% HI-FCS, 2 mM L-glutamine, 100 units/ml penicillin, 100 µg/ml streptomycin. *Sf9* cells were cultivated in IPL-41 medium (Genaxxon Bioscience) supplemented with 10% HI-FCS, 2% yeastolate, 1% lipid concentrate, 50 µg/ml gentamicin (Invitrogen), and 2.5 µg/ml amphotericin B (Genaxxon Bioscience). Detailed descriptions and references are available in Text S1.

### Cellular PdtIns(3,4,5)*P*_3_ Measurements

PtdIns(3,4,5)*P*_3_ levels have been measured as described in [9] with some modifications. BMMCs were cultured for 2 h in phosphate-free RPMI medium/2% FCS at 37°C and 5% CO_2_, followed by labelling with 1 mCi/ml [^32^P]-orthophosphate for 4 h. Cells were washed, stimulated, and lysed by the addition of chloroform/methanol (1∶2, v/v, with butylated hydroxytoluene and carrier phosphoinositides). Lipids were extracted, deacylated, and separated by high-pressure liquid chromatography (HPLC).

### In Vitro Lipid Kinase Assay

PI3Kγ-His_6_ was incubated with PtdIns(4,5)*P*_2_-containing lipid vesicles (PE/PS/PC/SM/PI*P*_2_ = 30/20/10/4.5/1.2; PI*P*_2_ final 5 µM), 10 µM ATP, and 4 µCi of [γ^32^P]-ATP in lipid kinase buffer (40 mM HEPES [pH 7.4], 150 mM NaCl, 4 mM MgCl_2_, 1 mM DTT [1,4-Dithio-DL-threitol], 0.1 mg/ml fatty-acid free BSA) for 10 min at 30°C. Alternatively, PtdIns/PS vesicles (∼200 µM each) were used. Reactions were terminated by addition of 1 M HCl and CHCl_3_/MeOH. Lipids were isolated by chloroform extraction, separated by TLC and quantified on a Typhoon 9400.

### Statistical Analysis

Numeric results were tested for significance using a two-tailed Student's *t* test, (paired or unpaired, as imposed by datasets). * or ^&^, ** or ^&&^, and *** or ^&&&^ refer to *p*-values *p*<0.05, *p*<0.005, and *p*<0.0005. * and ^&^ were used for comparison of different genotypes, stimuli an conditions as indicated. Calculations were carried out using Graph Pad Prism, Microsoft Excel, or Kaleidagraph software.

### Deuterium Exchange Reactions

Protein stock solutions (5 µl; Hsp110γ-C-His_6_: 30 µM; Hsp110γ-C-His_6_/Mmp84-C-His_6_: 35 µM) were prepared in 20 mM Tris [pH 7.5], 100 mM NaCl, 1 mM ammonium sulfate, and 5 mM DTT. Exchange reactions were initiated by addition of 25 µl of a 98% D_2_O solution containing 10 mM HEPES (pH 7.2), 50 mM NaCl, and 2 mM DTT, giving a final concentration of 82% D_2_O. Deuterium exchange reactions were allowed to carry on for seven time periods, 3, 10, 30, 100, 300, 1,000, and 3,000 s of on-exchange at 23°C, before addition of quench buffer. On-exchange was stopped by the addition of 40 µl of a quench buffer containing 1.2% formic acid and 0.833 M guanidine-HCl, which lowered the pH to 2.6. Samples were then immediately frozen in liquid nitrogen until mass analysis. The full HDX-MS protocol can be found in Text S1.

## Supporting Information

Figure S1 **PAF-mediated signaling does not activate PI3K, and does not synergize with FcεRI co-stimulation (related to** **Figure 1** **).** IgE-sensitized (100 ng/ml IgE, overnight) or non-sensitized wild type BMMCs were IL-3 depleted for 3 h and stimulated with either 1 µM adenosine (Ade), 1 µM PAF, or 5 ng/ml antigen (post IgE-sensitization) ± PAF for 2 min. Subsequently, cell lysates were subjected to SDS-PAGE. Phosphorylation of Ser473 in PKB/Akt (A), (B) Ser133 in cyclic AMP-responsive element-binding protein (CREB) and Ser660 in PKCβII was monitored by immunodetection with phosphosite-specific antibodies (*n* = 3, *: *p*<0.05; * refers to unstimulated control).
(EPS)

Figure S2 **Effect of PKC-inhibitors on PMA- or adenosine-induced PKB phosphorylation (S473) (related to** **Figure 3** **).** (A/B) Wild type BMMCs were starved for 3 h in IL-3 free medium supplemented with 2% FCS, and preincubated with the inhibitors for 20 min before stimulation (pan-PKC: Ro318425, Gö6983; classical PKC: PKC412 [CPG41251]; classical and atypical PKC: Gö6976; Rotterlin: broad band inhibitor; all 1 µM; & refers to comparison with untreated, stimulated control). (C) PKB activation in response to 100 nM PMA or 1 µM Thapsigargin (2 min) was analyzed in wild type, PKCα^−/−^ and PKCγ^−/−^ BMMCs. Cells were deprived of IL-3 as in (A/B). PKB (S473) and MAPK (T183/Y185) phosphorylation was determined by Western blotting. (D) Wild type, PKCβ^−/−^ and PI3Kγ^−/−^ BMMCs were stimulated with different concentrations of thapsigargin, before degranulation was quantified by the measurement of β-hexosaminidase release into cell supernatants (data are the average of three independent experiments ± standard error of the mean [SEM]; * comparison to wild type (both genotypes); & only the p110γ^−/−^ dataset reached significance).
(EPS)

Figure S3 **Identification of PI3Kγ phosphorylation sites by MS (related to** **Figure 4** **).** (A/B) Recombinant, catalytically inactive GST-PI3Kγ (K833R mutant; GST fused to p110γ amino acids 38–1,102) was phosphorylated in vitro by recombinant PKCβ in the presence of 100 µM ATP/[γ^32^P]-ATP. Proteins were separated by SDS-PAGE and trypsin-digested PI3Kγ was analyzed by LC-MSMS. (A) Enhanced product ion spectra of the tryptic phospho-S582-peptide of PI3Kγ. The y- and b-fragments detected are indicated in the sequence. Fragments showing a H_3_PO_4_ loss are marked with an asterisk. The b_2_, y_6_, and y_7_ fragments allow assignment of the phosphorylation to serine 3 in the peptide. (B) Enhanced product ion spectra of the non-phosphorylated form of this peptide. (C) Relevant information for the MRM analysis of the peptides containing Ser582. The amino acid numbering is as in Swiss-Prot entry P48736. (D) Sequence alignment of the beginning of the helical domain of class I PI3Ks. Alignments were done by inspection of the crystal structures of PI3Kγ (1E8Y), PI3Kα (3HHM), PI3Kβ (2Y3A), and PI3Kδ (2WXR). Secondary structure elements are labeled as indicated in the legend. S582 is colored red, while cancer-associated PI3Kα mutations are marked as blue.
(TIF)

Figure S4 **Anti-phospho-Ser582 antibody validation (related to** **Figure 5** **).** (A) Wild type BMMCs were transfected with empty vector, expression plasmid for GFP-PI3Kγ wild type or the GFP-PI3Kγ S582A mutant. On the next day, cells were stimulated with 200 nM PMA for 45 s, and PI3Kγ was immunoprecipitated from cell lysates. Specificity of the anti-S582 antibodies was validated by Western blotting.
(EPS)

Figure S5 **Global and site-specific in vitro phosphorylation of monomeric p110γ and p110γ-p84 complexes by PKCβ and CamKII (related to** **Figure 5** **).** (A) Equal amounts of purified recombinant p110γ-His_6_ (2.5 pmole) or p110γ-His_6_/EE-p84 complexes were incubated with 20 pmole wortmannin to eliminate auto-phosphorylation signals. Free or complexed p110γ (with p84 protein) was incubated with 10 µM ATP and 5 µCi of [^32^P]-γ-ATP, and equal specific activity of recombinant PKCβII and CamKII (Life Technologies assays: 30 pmol phosphate incorporation/min.) for 30 min at 30°C. Subsequently, proteins were denatured and separated by SDS-PAGE followed by Coomassie staining (lower panel). ^32^P-incorporation was visualized by autoradiography and quantified on a phospho-imager (Typhoon 9400, middle panel). Band intensities were quantified with ImageQuant TL Software (Amersham Biosciences, top panel; *n* = 4, * *p*<0.05). Insert: quantification of p84 phosphorylation from the reactions shown in (A), (open bars, *n* = 4, * *p*<0.05). Phospho-PKCβII levels were subtracted from phospho-p84 signals (due to identical apparent Mr on SDS-PAGE). The filled bar represents phosphorylated p84-His_6_ after Ni^2+^-NTA pull-down to minimize contaminating PKCβII autophosphorylation signals (*n* = 3). (B) Equal amounts of purified recombinant p110γ-His_6_ (2.5 pmole) or p110γ-His_6_/EE-p84 complexes were incubated as indicated with ATP (100 µM), recombinant PKCβII and CamKII for 30 min at 30°C. Proteins were denatured, separated by SDS-PAGE followed by immunological detection of p110γ and p84 protein, as well as site-specific phosphorylation of residue Ser582 (pp110γ S582) in p110γ. PKCβII and CamKII input was adjusted to equal protein kinase activity (30 pmol of transferred phosphate/min). For quantification, Ser582 phosphorylation levels were normalized (*n* = 4, *: *p*<0.05, ***: *p*<0.0005).
(EPS)

Figure S6 **Phosphorylation of p110γ requires active PKCβ (related to** **Figure 5** **).** (A) Effect of PKC inhibitors on thapsigargin induced p110γ Ser582 phosphorylation. IL-3 deprived BMMCs were preincubated with PKC inhibitors (1 µM Ro318425 or 1 µM AEB071) for 20 min before stimulation with 1 µM thapsigargin for 2 min. PI3Kγ was immunoprecipitated from cell lysates with anti-p110γ antibody (see Methods). Precipitated protein was then probed for phosphorylated p110γ (pp110γ) using phospho-specific anti-pSer582 antibodies. PI3Kγ phosphorylation is shown normalized to total p110γ levels (mean ± standard error of the mean [SEM], *n* = 3; * depict comparison with stimulated control). (B) Cells were stimulated as in (A). PKB (T308) and CamKII (T286) phosphorylation was determined by Western blotting. Data are the average of three independent experiments ± SEM.
(EPS)

Figure S7 **Ser582 phosphorylation releases p84 from p110γ (related to** **Figure 6** **).** IL-3 deprived BMMCs were stimulated with 100 nM PMA for 2 min. PI3Kγ complex was co-immunoprecipitated from cell lysates using either (A) anti-p110γ or (B) anti-p84 antibodies. The amount of p84 co-immunoprecipitated with p110γ (A) or p110γ co-immunoprecipitated with p84 (B) was normalized to the total amount of p110γ or p84, respectively. Data are the average of five independent experiments ± standard error of the mean [SEM].
(EPS)

Figure S8 **PI3Kγ domain order and peptide coverage after pepsin digestion (related to** **Figure 7** **).** Identified and analyzed peptides are shown under the primary sequence of PI3Kγ, which has been colored according to the domain boundaries shown above.
(EPS)

Figure S9 **Changes in deuteration levels of PI3Kγ in the presence of p84 (related to** **Figure 7** **).** Peptides spanning PI3Kγ (labeled A–Q) that showed greater than 0.5 Da changes in deuteration in the presence and absence of p84 were mapped onto the PI3Kγ structure (PDB ID: 2CHX, residues 144–1,093). The greatest difference in exchange observed at any time was used for the mapping. S582 is shown as red balls. The ATP competitive inhibitor PIK-90 is shown in green as a reference point for the kinase domain. The linker regions between the RBD and the C2 domain and the C2 and helical domain are shown as dotted lines.
(EPS)

Figure S10 **Changes in deuteration levels of PI3Kγ peptides in the presence of p84 (related to** **Figure 7** **).** The graphs showing the number of incorporated deuterium atoms in the presence (o) and absence (•) of p84 at seven time points in peptides that showed a greater than 0.5 Da H/D exchange difference. Data represent mean ± standard deviation (SD) of two independent experiments.
(EPS)

Figure S11 **Deuteration levels in free and p84-bound p110γ (related to** **Figure 7** **).** Changes in deuteration levels were mapped onto the crystal structure of PI3Kγ (PDB ID: 2CHX) as in Figure 7.The isotopic profiles of two selected peptides (579–592, 623–630) from the helical domain are shown at three or four time points of H/D on exchange +/− the p84 subunit. In the absence of the p84 adaptor the majority of peptides in the helical domain showed broadening of the isotopic profiles indicative of EX1 kinetics (see 30 s of HDX in free p110γ). The helices HB1, HA2 (579–592), and HA3 (624–631) selected are all structurally linked, with HA3 located at the interface of the helical domain with the C-lobe. Ser582 (red) and Thr1024 (yellow) have been highlighted as a reference.
(TIF)

Table S1 **Deuterium exchange data of all analyzed peptides of PI3Kγ in the absence or presence of p84 are summarized in tabular form (related to** **Figure 7** **).** Percent hydrogen deuterium exchange was calculated for each of the seven time points and colored according to the legend. Data show the mean of two independent experiments. The charge state (Z), maximal number of exchangeable amides (#D), starting residue number (S), and ending residue number (E) are displayed for every peptide.
(XLSX)

Text S1 **Extended experimental procedures, reference to animals and plasmids.** Detailed description of experimental procedures, materials, and further reference to the origin of genetically modified mice used here, and a primer to the determination of deuterium incorporation (HDX_MS).
(DOCX)

## Abbreviations

A3AR: A3 adenosine receptor
ADA: adenosine deaminase
BAPTA-AM: 1,2-Bis(2-aminophenoxy)ethane-N,N,N′,N′-tetraacetic acid tetrakis(acetoxymethyl ester)
BMMC: bone marrow-derived mast cell
CAMK: calmodulin-dependent kinase
FcεRI: high-affinity IgE receptor
GPCR: G protein-coupled receptor
GST: glutathione S-transferase
HDX-MS: hydrogen deuterium exchange mass spectrometry
MRM: multiple reaction monitoring
PAF: platelet activating factor
PI3K: phosphoinositide 3-kinase
PKC: protein kinase C
PMA: phorbol 12-myristate 13-acetate
PtdIns: phosphatidylinositol
PKB/Akt: protein kinase B
PTx: *B. pertussis* toxin
SCF: stem cell factor, c-kit ligand
SOCE: store-operated Ca2+ entry
