# Supplementary figures and images for: PKCβ Phosphorylates PI3Kγ to Activate It and Release It from GPCR Control
Source: PLoS Biol. 2013 Jun 25;11(6):e1001587. doi: 10.1371/journal.pbio.1001587 (PMC3692425; doi:10.1371/journal.pbio.1001587)

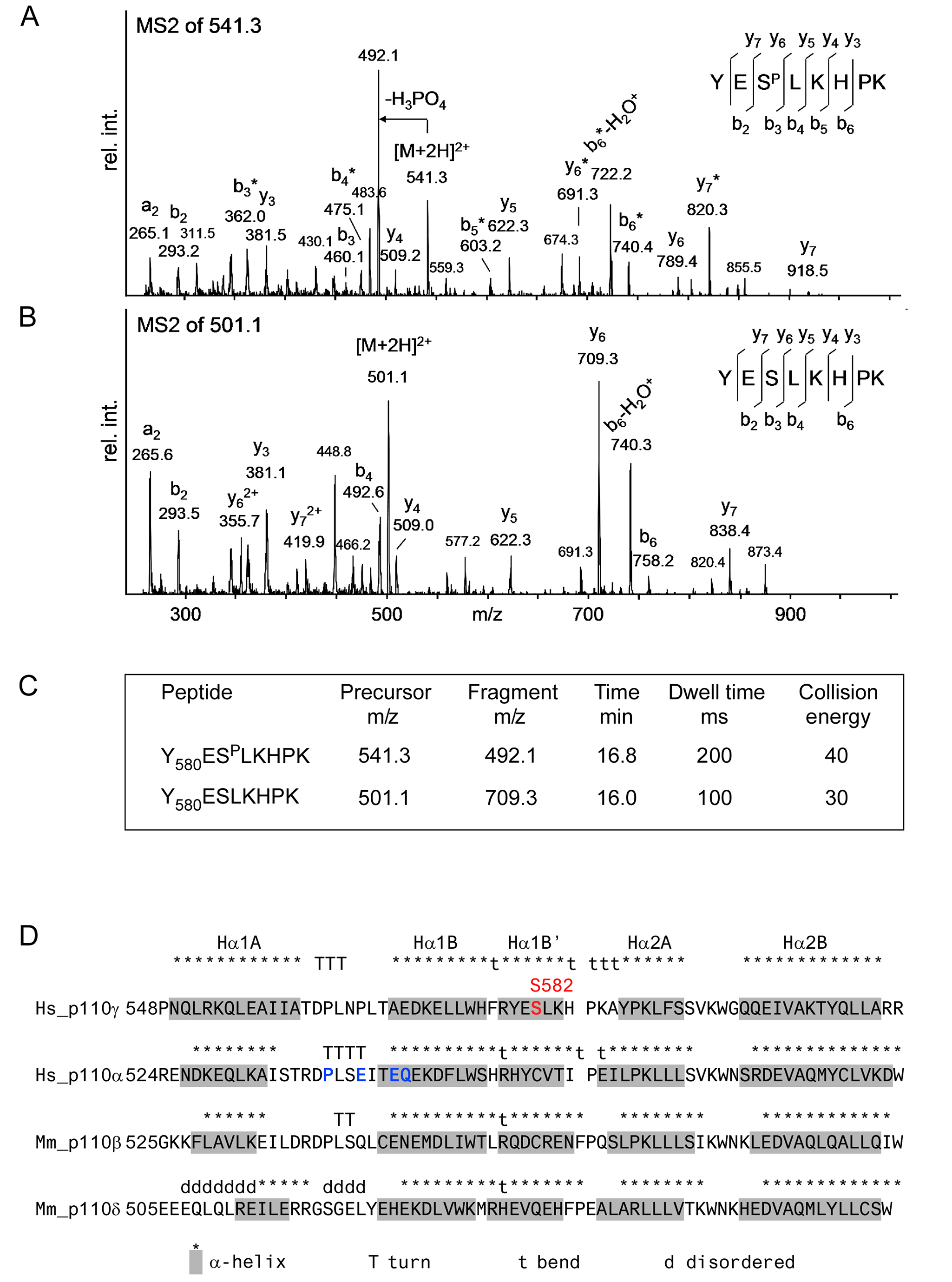

Supplement: Figure S3 — Identification of PI3Kγ phosphorylation sites by MS (related to Figure 4 ). (A/B) Recombinant, catalytically inactive GST-PI3Kγ (K833R mutant; GST fused to p110γ amino acids 38–1,102) was phosphorylated in vitro by recombinant PKCβ in the presence of 100 µM ATP/[γ32P]-ATP. Proteins were separated by SDS-PAGE and trypsin-digested PI3Kγ was analyzed by LC-MSMS. (A) Enhanced product ion spectra of the tryptic phospho-S582-peptide of PI3Kγ. The y- and b-fragments detected are indicated in the sequence. Fragments showing a H3PO4 loss are marked with an asterisk. The b2, y6, and y7 fragments allow assignment of the phosphorylation to serine 3 in the peptide. (B) Enhanced product ion spectra of the non-phosphorylated form of this peptide. (C) Relevant information for the MRM analysis of the peptides containing Ser582. The amino acid numbering is as in Swiss-Prot entry P48736. (D) Sequence alignment of the beginning of the helical domain of class I PI3Ks. Alignments were done by inspection of the crystal structures of PI3Kγ (1E8Y), PI3Kα (3HHM), PI3Kβ (2Y3A), and PI3Kδ (2WXR). Secondary structure elements are labeled as indicated in the legend. S582 is colored red, while cancer-associated PI3Kα mutations are marked as blue. (TIF) [file pbio.1001587.s003.tif]

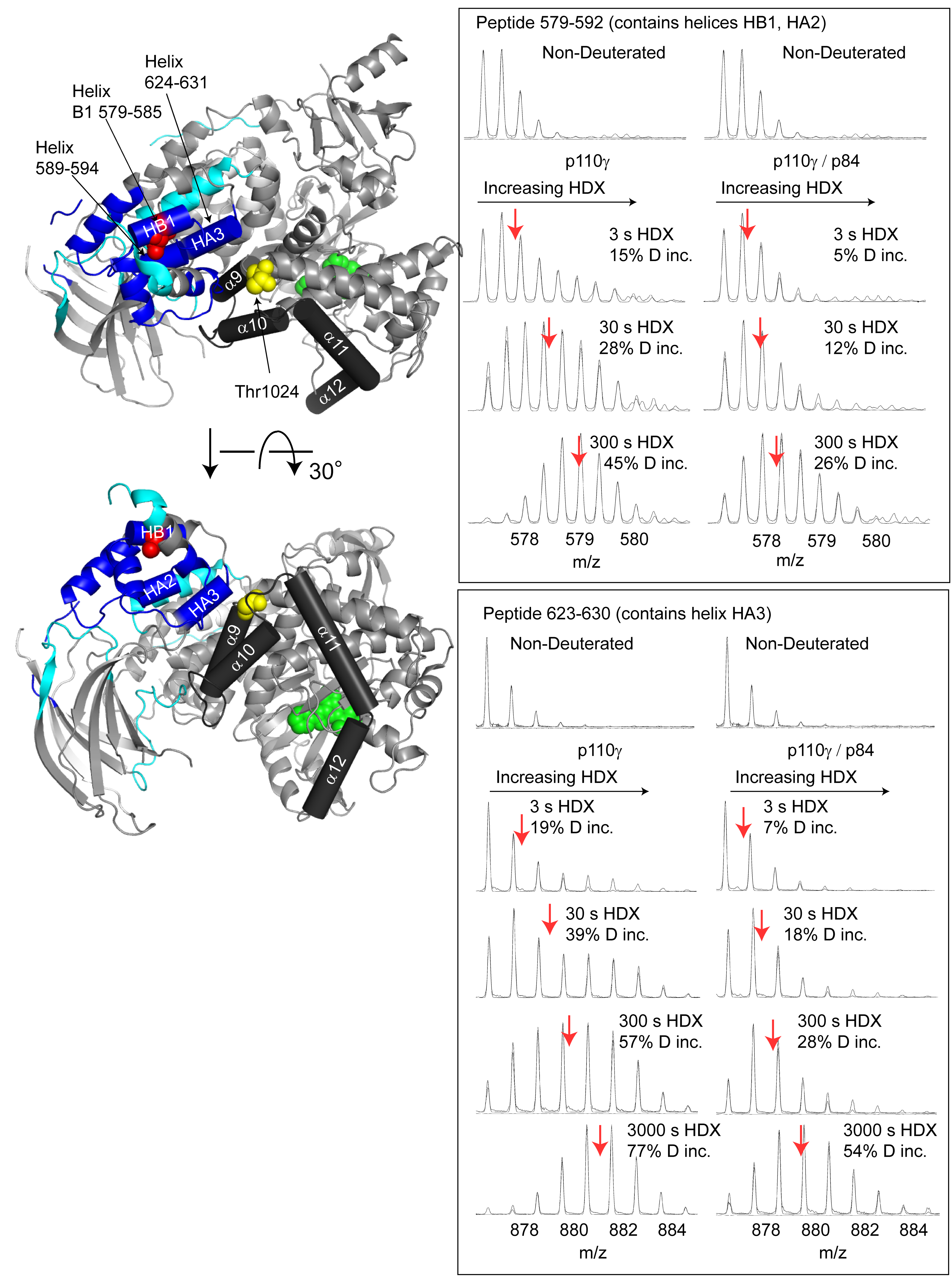

Supplement: Figure S11 — Deuteration levels in free and p84-bound p110γ (related to Figure 7 ). Changes in deuteration levels were mapped onto the crystal structure of PI3Kγ (PDB ID: 2CHX) as in Figure 7.The isotopic profiles of two selected peptides (579–592, 623–630) from the helical domain are shown at three or four time points of H/D on exchange +/− the p84 subunit. In the absence of the p84 adaptor the majority of peptides in the helical domain showed broadening of the isotopic profiles indicative of EX1 kinetics (see 30 s of HDX in free p110γ). The helices HB1, HA2 (579–592), and HA3 (624–631) selected are all structurally linked, with HA3 located at the interface of the helical domain with the C-lobe. Ser582 (red) and Thr1024 (yellow) have been highlighted as a reference. (TIF) [file pbio.1001587.s011.tif]
