# Supplementary material for: PKCβ Phosphorylates PI3Kγ to Activate It and Release It from GPCR Control
Source: PLoS Biol. 2013 Jun 25;11(6):e1001587. doi: 10.1371/journal.pbio.1001587 (PMC3692425; doi:10.1371/journal.pbio.1001587)
Supplement: Text S1 — Extended experimental procedures, reference to animals and plasmids. Detailed description of experimental procedures, materials, and further reference to the origin of genetically modified mice used here, and a primer to the determination of deuterium incorporation (HDX_MS). (DOCX) [file pbio.1001587.s013.docx]

# SUPPLEMENTAL INFORMATION

**PKCβ Phosphorylates PI3Kγ to Activate it and Release it from GPCR Control**

Romy Walser^1^, John E. Burke^2^, Elena Gogvadze^1^, Thomas Bohnacker^1^, Xuxiao Zhang^2^, Daniel Hess^3^, Peter Küenzi^1^, Michael Leitges^4^, Emilio Hirsch^5^, Roger L. Williams^2^, Muriel Laffargue^6^, Matthias P. Wymann^1,*^

^1^Department of Biomedicine, University of Basel, 4058 Basel, Switzerland;

^2^Medical Research Council, Laboratory of Molecular Biology, Cambridge CB2 0QH, UK;

^3^Friedrich Miescher Institute for Biomedical Research, Basel 4058, Switzerland;

^4^Biotechnology Centre, University of Oslo, 0349 Oslo, Norway.

^5^Department of Genetics, Biology and Biochemistry, University of Torino, Torino 10126, Italy;

^6^INSERM U563, Département Lipoprotéines et Médiateurs Lipidiques, Toulouse 31300, France

**Correspondence*: Matthias.Wymann@UniBas.CH

Matthias P. Wymann

Inst. Biochemistry & Genetics

Dept. Biomedicine

University of Basel

Mattenstrasse 28

4058 Basel, Switzerland

## EXTENDED EXPERIMENTAL PROCEDURES

## Materials

Where not indicated otherwise, materials and chemicals were purchased from Sigma-Aldrich.

## Mice

C57BL6/J wild type (Jackson Laboratories), p110γ^-/-^ [1], PKCα^-/-^ [2,3], PKCβ^-/-^ [4], and PKCγ^-/-^ [5] mice were used for the preparation of BMMCs.

## Cell Culture

Mouse bone marrow-derived mast cells (BMMCs) were developed from progenitor cells of femurs of 8-12 week old mice on a pure C57BL/6J genetic background [6]. Femurs were cut off at both ends and the marrow collected by centrifugation. Cells were resuspended in complete growth medium: Iscove’s modified Dulbecco’s medium (IMDM) supplemented with 10% heat-inactivated fetal calf serum (HI-FCS), 2 mM L-glutamine, 100 units/ml penicillin, 100 μg/ml streptomycin, 50 μM β-mercaptoethanol, 2 ng/ml recombinant murine IL-3 (Peprotech) and initially 1x 5 ng/ml SCF, and cultured at 37°C, 5% CO_2_. IL-3 was re-added every third day. Non-adherent cells were transferred to new flasks and diluted weekly to 0.5 M cells/ml using 80% of fresh and 20% of conditioned medium. Differentiation of BMMCs was confirmed by measuring expressing of c-Kit and IgE receptors by flow cytometry. All mast cell experiments were carried out with BMMCs cultured for 4 to max. 16 weeks.

The human embryonic kidney 293 (HEK293) cell line was grown in Dulbecco’s modified essential medium (DMEM) supplemented with 10% HI-FCS, 2 mM L-glutamine , 100 units/ml penicillin, 100 μg/ml streptomycin at 37°C, 5% CO2.

Spodoptera frugiperda *(*Sf9) cells were grown in IPL-41 medium (Genaxxon Bioscience) supplemented with 10% HI-FCS (Sigma-Aldrich), 2% yeastolate, 1% lipid concentrate, 50 μg/ml gentamicin (all Invitrogen) and 2.5 μg/ml amphotericin B (Genaxxon Bioscience). Sf9 cells were cultured as adherent or suspension culture at 27°C. Suspension cells were grown in Erlenmeyer flasks in a shaking incubator at 75-90 rpm.

## Plasmids

Full-length, untagged human PI3Kγ was expressed from pcDNA3 (Invitrogen) [7]. Point mutations were introduced by polymerase chain reaction (PCR) using the overlap extension technique [8]. The mouse PKCβ2 cDNA was kindly obtained from Jae-Won Soh (PKClab.org) and inserted BamHI-BstEII-XhoI into pcDNA3 containing a Kozak translation initiation sequence and the human influenza hemagglutinin (HA)-tag between HindIII and BamHI (pcDNA3-kzHA). The catalytic part of PKCβ2 (PKCβ2-CAT) including amino acids 302-673, was amplified from full-length PKCβ2 with the forward primer 5’-ccgcaGGATCCcagaagtttgagagagccaagattg and the reverse primer 5’-ggcgTCTAGAttagctcttgacttcaggttttaaaaattc and inserted BamHI-XbaI into pcDNA3-kzHA. The pseudosubstrate deletion mutant of PKCβ2 was produced by the overlap extension technique with primers lacking the coding sequence for amino acids 19-31 of PKCβ2 (forward: 5’-gagcacagtgcacgaggtgaagaaccacaaattcac, reverse: 5’-cacctcgtgcactgtgctctcctcgccc) and two helper primers. The baculovirus transfer plasmid encoding GST-PI3Kγ (pAcG2T-PI3Kγ, codons 38-1102) has been described [9]. The transfer plasmid for baculovirus-mediated expression of C-terminal His-tagged full-length human PI3Kγ has been derived from pVL1393 (Invitrogen) containing codons 144-1102 of human PI3Kγ [10]) by inclusion of codons 1-143 by PCR. Serine 582 mutations were introduced into this construct by insertion of a PciI-EcoRI or BstEII-EcoRI fragment from mutant PI3Kγ in pcDNA3, respectively. All constructs were confirmed by sequencing.

**Transfections**

HEK293 cells were seeded into 6 cm dishes and were transfected with JetPEI^TM^ (Polyplus-transfection) using 2.5 μg of total plasmid DNA. BMMCs were transfected by nucleofection (Amaxa) using solution T, 1-3 μg of PI3Kγ and, if indicated, 0.2-2 μg of p84 expression plasmid. Total DNA concentration was adjusted to 10 μg with pcDNA3 vector.

**Stimulation of BMMCs**

BMMCs were incubated overnight with 100 ng/ml anti-dinitrophenyl (DNP)-specific immunoglobulin E (clone SPE-7) in complete growth medium (see cell culture, above). Next day, cells were centrifuged for 5 min at 146 g and resuspended in IL-3 free growth medium containing 2% (signaling experiments) or 10% FCS (degranulation assay). Degranulation was induced by the addition of DNP coupled to human serum albumin (DNP-HSA) at the indicated concentrations. Other stimuli used to trigger BMMC activation were thapsigargin, phorbol 12-myristate 13-actetate (PMA; 100nM, Alexis), adenosine (1 μM), *N*^6^-(3-iodobenzyl)-adenosine-5’-*N*-methylcarbox-amide (IB-MECA; 10 nM), stem cell factor (SCF; 10 ng/ml, Peprotech), interleukin-3 (IL-3; 10 ng/ml, Peprotech), and platelet activating factor (PAF [1 uM], β-Acetyl-γ-O-alkyl-L-α-phosphatidylcholine). Stimulations were done at 37°C. Preincubation with *B. Pertussis Toxin* (PTx; 100 ng/ml) was done for 4 hours, and adenosine deaminase (ADA; 10 units/ml) was added 1 min before stimulation. Inhibitor pretreatment time was 15 min for wortmannin (100 nM), and 20 min for the PKC inhibitors (Ro318425 (1 μM), Gö9683 (0.5 μM), Gö6976 (0.5 μM), and Rottlerin (10 μM), a brad-band kinase inhibitor [11] [all from Calbiochem]; PKC412/CPG41251 (1 μM, LC Laboratories), Sotrastaurin (AEB071; 1μM, Selleckchem). For Western blot analysis of PKB and MAPK phosphorylation, stimulations were stopped on ice, and cells were collected by centrifugation for 1 min at 2000 g (4°C) and immediately lysed in 1x Laemmli sample buffer [62.5 mM Tris/HCl pH 6.8, 2% SDS, 10% glycerol, 5% β-mercaptoethanol, bromophenolblue].

**(Co-)Immunoprecipitation**

Transfected HEK293 cells were washed with phosphate buffered saline (PBS) and lysed with NP-40 lysis buffer [50 mM Tris/HCl pH 7.4, 138 mM NaCl, 2.7 mM KCl, 5% glycerol, 1% NP-40] supplemented with protease inhibitors [20 μM leupeptin, 18 μM pepstatin, 5 μg/ml aprotinin, phenylmethylsulfonylfluorid (PMSF)], phosphatase inhibitors [40 mM NaF, 2 mM Na_3_VO_4_], 0.5 mM EDTA and 0.5 mM EGTA for 15 min on ice. Insoluble material was pelleted by centrifugation and one-tenth of clarified lysate was mixed with Laemmli sample buffer to examine overall protein expression. The remainder of lysate was incubated with anti-p110γ (ascites fluid, 641, corresponds to Jena Bioscience ABD-027) or anti-HA.11 (Covance) antibodies for 1 hour at 4°C on a rotating wheel, followed by incubation with 25 μl of a 50% slurry of GammaBind Plus Sepharose (Amersham Biosciences) or protein G agarose (Millipore) for additional 2 hours. Immune complexes were washed 3x with NP-40 lysis buffer, 1x with 0.1 M Tris/HCl pH 7.4, 0.5 M LiCl, 0.2% NP-40, 1x again with NP-40 lysis buffer, and eluted by the addition of Laemmli sample buffer.
BMMCs were collected by centrifugation and lysed in NP-40 lysis buffer supplemented with protease inhibitors, phosphatase inhibitors [40 mM NaF, 10 mM β-glycerophosphate, 5 mM sodium pyrophosphate], 2 mM EDTA and 2 mM EGTA for 15 min on ice. Cell debris were removed by centrifugation, and endogenous or transfected PI3Kγ was immunoprecipitated from the cleared lysate with anti-PI3Kγ (ascites fluid, clone 641) antibodies (1-2 hours) and protein A or G-agarose beads (2-5 hours). Immune complexes were washed as describe above before resuspension in 1x Laemmli sample buffer.
 For co-immunoprecipitations from BMMCs, cells were suspended in hypotonic lysis buffer (20 mM Tris/HCl pH 7.4, 20 mM NaCl, 2.7mM KCl, 5% glycerol, 0.5% CHAPS) supplemented with protease inhibitors [20 μM leupeptin, 18 μM pepstatin, 5 μg/ml aprotinin, 1mM phenylmethylsulfonylfluorid (PMSF), 0.5mM diisopropyl fluorophosphate], phosphatase inhibitors [40 mM NaF, 10 mM β-glycerophosphate, 5 mM sodium pyrophosphate], 2 mM EDTA and 2 mM EGTA. PI3Kγ complex was immunoprecipitated from cleared cell lysates with either anti-p110γ (ascites fluid, clone 641) or anti-p84 (goat antisera, for epitopes see [12]) antibodies for 1 h, before protein G-agarose beads were added for another hour. Beads were washed three times with lysis buffer and resuspended in 1x Laemmli sample buffer. Lysates and immunoprecipitations were analysed by Western blotting.

## Western Blotting

Protein samples were heated to 95°C for 5 min, centrifuged for 2 min at 16’000g, separated by SDS-polyacrylamide gel electrophoresis (PAGE) and transferred to polyvinylidene fluorid (PVDF)-membranes (Millipore) by semi-dry transfer. Membranes were blocked with 5% (w/v) milk powder (Migros, Schweiz) in TBS-T buffer [20 mM Tris/HCl, pH 7.6, 137 mM NaCl, 0.1% (v/v) Tween 20 (Fluka)]. Blots were incubated overnight at 4°C with the primary antibody, followed by incubation with horseradish peroxidase (HRP)-coupled secondary goat anti-mouse or anti-rabbit antibodies. Protein bands were visualized by enhanced chemiluminescence (ECL) (Millipore). Phospho-specific antibodies were kept in TBS-T/1% BSA [pPKB S473, pPKB T308, pCamKII T286, pCREB S133 and pan-phospho PKC (S660 in PKCβII; all from Cell Signaling); pMAPK (Sigma M8159 or Promega V8031); phospho-p110γ S582 antibodies were raised against Cys-conjugated peptides C+KELLWHFRYE-[phosphoSer]-LKHPKAYPKLFSS and C+WHFRYE-[phosphoSer]-LKHPK, and affinity purified]. All other antibodies were diluted in TBS-T/1% (w/v) dry milk powder [PI3Kγ (raised against amino acids 97-335); PKCβ2 (Santa Cruz, sc-210), PKCα (sc-208), PKCγ (sc-211), β-Tubulin (Boehringer Mannheim), α-Tubulin (Sigma, T9026), HA (Covance, MMS-101R), p84 (rabbit antisera, [12]), total PKB (a kind gift from B. Hemmings)]. Quantifications of Western blots have been done with ImageJ software (NIH).

## β-Hexosaminidase Release Assay

Mast cell degranulation was assessed by the release of β-hexosaminidase into cell supernatants [13,14]. BMMCs were cultured at 0.8-1 Mio cells/ml in complete growth medium, and were, pre-loaded with 100 ng/ml IgE (for IgE/Ag experiments). Cells were wash once and resuspended in PIPES-buffered solution [25 mM PIPES pH 7.4, 119 mM NaCl, 5 mM KCl, 5.6 mM glucose, 1 mM CaCl_2_, 0.4 mM MgCl_2_, 0.1 % fatty-acid free, low endotoxin bovine serum albumin (LE-BSA, Sigma)]. Cells (0.5 M) were stimulated with IgE/DNP-HSA or thapsigargin in a total volume of 500 μl PIPES-buffered solution in 24 well plates. Following incubation at 37°C for 20 min, plates were centrifuged at 4°C at 330 g for 5 min. Then, 100 μl aliquots of supernatant were transferred to 96 well plates. To determine total β-hexosaminidase, unstimulated cells were lysed by the addition of PIPES-buffered solution with 0.1% Triton X-100 for 10 min, and 100 μl aliquots were transferred to 96 well plates. Activity of β-hexosaminidase was assessed by incubation with 100 μl of substrate solution [100 mM p-nitrophenyl-N-acetyl-β-D-glucosaminidine (p-NAG) in 0.1 M sodium acetate pH 4] for 1 h at 37°C. The reaction was stopped with 100 μl of 0.15 M Na_2_CO_3_/NaHCO_3_, and the amount of produced p-nitrophenol was assessed by the absorbance at 410 nm. Background values were obtained from lysates of unstimulated cells incubated without p-NAG. The amount of β-hexosamidinase released was calculated by using the formula: Release (R) [%] = (A_Stimulated_ – A_Unstimulated_)/(A_Total unstimulated_ – A_Total background_)*100

**Cytosolic Ca^2+^ concentration measurements**

BMMCs were suspended in physiologic HEPES buffer (10 mM HEPES/NaOH pH 7.5, 137 mM NaCl, 2.7 mM KCl, 5 mM glucose, 1 mM CaCl_2_, 1 mM MgCl_2_, 0.1 % fatty acid free BSA]. Cells were loaded with 4 μM Fura-4F/AM [K_D_ for Ca^2+^ *in vitro* is 770 nM; intracellular K_D_ for Ca^2+^ is ca. 1 µM [15,16]) for 10 min, washed twice, and resuspended in HEPES buffer containing different Ca^2+^ concentrations ([Ca^2+^]_e (extracellular)_ = 0 - 1 mM). Cells were subsequently stimulated in final volume of 3 ml (0.5 M cells/ml) in a continuously stirred cuvette at 37° in a fluorescence spectrometer (Perkin Elmer LS50B). The excitation wavelength was alternated between 340 and 380 nm and fluorescence emission was measured at 510 nm every 200 ms. After recording a base line cells were stimulated (e.g. with 0.5 μM thapsigargin). To calculate [Ca^2+^]_i_ maximal Fura fluorescence (F_max_) was determined after cell permeabilisation with 0.1% Triton X-100 after addition of 2 mM Ca^2+^. Minimal fluorescence (F_min_) values were obtained after the addition of 4 mM EGTA. Autofluorescence was subtracted before [Ca^2+^]_i_ was calculated using the equation of [17]:

[Ca^2+^]_i_ = K_d_[(R - R_min_)/(R_max_ - R)](F_380 EGTA_/F_380 Triton_), R = F_340_/F_380_

To correlate intracellular Ca^2+^ concentrations with PKB phosphorylation, [Ca^2+^]_i_ levels were plotted against relative levels of phosphorylated PKB (S473). Phospho-PKB levels were obtained from cells (1.5 ml) taken out of the measurement cuvette 2 min after stimulation. Lysed cells were analyzed by Western blotting and PKB phosphorylation was quantified by intensity measurements with ImageJ software.

**Production of recombinant PI3Kγ**

Recombinant human PI3Kγ was expressed as N-terminal GST- or C-terminal His_6_-tagged-fusion protein in Sf9 cells by infection with recombinant baculovirus. Plasmid (pAcG2T-PI3Kγ, codons 38-1102) and baculovirus for GST-PI3Kγ have been described [9]. Full-length PI3Kγ-His_6_ and S582 point mutants were expressed in pVL1393 (BD Biosciences) after co-transfection of Sf9 cells with BaculoGold DNA (BD Biosciences). Proteins were harvested as described in [18]. Baculovirus-infected Sf9 cells were harvested 2-2.5 days postinfection, were washed with PBS und lysed with either GST- or His-lysis buffer [GST: 50 mM Tris/HCl pH 7.5, 150 mM NaCl, 5 mM EDTA, 1 mM NaF, 1 mM DTT, 1% Triton-X100; His: 50 mM NaH_2_PO_4_·2H_2_O/NaOH, 300 mM NaCl, 20 mM imidazol, 5% glycerol, 1% NP-40] containing protease inhibitors. GST-PI3Kγ was purified on glutathione sepharose 4B (Amersham Biosciences) and eluted with 50 mM Tris/HCl pH 7.5, 150 mM NaCl, 5 mM EDTA, 1 mM DTT, 5% glycerol, 20 mM glutathione after extensive washing. PI3Kγ-His_6_ was purified over Ni^2+^-NTA agarose (Qiagen) and eluted with 50 mM NaH_2_PO_4_·2H_2_O/NaOH pH 8, 300 mM NaCl, 250 mM imidazol. Proteins were mixed 1:1 with 2x storage buffer [80 mM HEPES/NaOH, 4 mM EDTA, 2 mM DTT, 20 mM benzamidine, ~90% ethylenglycol] and kept at -20°C. Protein concentrations were quantified by Coomassie-staining in SDS-PAGE gels using the Odyssey infrared imaging system (LI-COR Biosciences). Bovine serum albumin served as standard.

## In vitro kinase assay

Recombinant GST-PI3Kγ wild type (wt) or kinase inactive mutant (KR, Lys833 mutated to Arg) was incubated at an equal molar ratio (if not stated differently) with recombinant PKCβ2 (40 ng, Invitrogen) in 1x PKC kinase buffer (50 mM HEPES pH 7.4, 10 mM MgCl_2_, 1 mM CaCl_2_, 1 mM DTT, 0.03% Triton X-100) in the presence of 10 μM adenosine 5’-triphosphate (ATP) and 5 μCi of [γ^32^P]-ATP [Perkin Elmer, 6000 Ci/mmol] per sample in a final volume of 40 μl. Ca^2+^/calmodulin-dependent protein kinase II (CamKII, Life Technologies)-mediated phosphorylation was performed in 1x CamKII kinase buffer (10 mM HEPES, 10 mM MgCl_2_, 1 mM Na_3_VO_4_, 10 ug/ml calmodulin, 0.5 mM CaCl_2_, 5 mM DTT). Kinase reactions were pre-warmed for 2 min at 30°C, started by the addition of 4 μl of ATP-mix (in H_2_O), and incubated at 30°C for 30 min. Reactions were terminated by the addition of 5x sample buffer and the proteins were resolved by SDS-PAGE. Gels were stained with Coomassie brilliant blue (Serva blue G); ^32^P-incorporation was visualized by autoradiography or quantified on a phosphoimager (Typhoon 9400). Band intensities were quantified with ImageQuant TL Software (Amersham Biosciences). A dilution series of the ATP/[γ^32^P]-γ-ATP-mix served as a standard to calculate absolute ^32^P_i_-incorporation rates.

Where indicated, phosphatidylserine (PS) lipid vesicles (0.1 mg/ml) containing 1-oleoyl-2-acetyl-sn-glycerol (OAG, 0.01 mg/ml, Cayman Chemical) were added. PS in CHCl_3_/MeOH 2:1 was mixed with OAG in acetonitril in an Eppendorf tube, dried by a stream of nitrogen gas and resuspended in kinase buffer by sonication.

## In vitro lipid kinase assay

Assays were performed in a final volume of 50 μl in 1.5 ml Eppendorf tubes in lipid kinase buffer [40 mM HEPES, pH 7.4, 150 mM NaCl, 4 mM MgCl_2_, 1 mM DTT, 0.1 mg/ml fatty-acid free BSA] in the presence of PtdIns(4.5)*P*_2_-containing lipid vesicles, 10 μM adenosine 5’-triphosphate (ATP) and 4 μCi of [γ^32^P]-ATP [Perkin Elmer, 6000 Ci/mmol] per sample. Phospholipid composition of the lipid vesicles has been chosen to mimic relative mole ratios found in the inner leaflet of the plasma membrane (PE/PS/PC/SM/PI*P*_2_ = 30/20/10/4.5/1.2-4.6). Lipids dissolved in CHCl_3_/MeOH (2:1) were combined in a glass tube [f. c. in assay: 130 μM PE, 87 μM PS, 43 μM PC, 20 μM SM, 5-20 μM PI*P*_2_], dried under a stream of nitrogen gas and resuspended in lipid kinase buffer without BSA by sonication (3x 15 seconds at an amplitude of 30%) on ice with a tip sonicator (U200S, IKA Labortechnik). Recombinant wild type or mutant PI3Kγ-His6 (200 ng/reaction) were diluted in lipid kinase buffer and added (8 μl) to 4 μl of mastermix [lipid kinase buffer, but with 1.05 mg/ml BSA] followed by the addition of 30 μl of lipid mix. Samples were mixed and kept on ice for additional 20 min, were then warmed up to 30°C for 2 min on a heat block before starting the reaction by the addition of 8 μl of ATP-mix in lipid kinase buffer without BSA. Samples were incubated for 10 min at 30°C with shaking (1200 min^-1^) on a heat block. The reaction was stopped by the addition of 100 μl 1 N HCl and the lipids were extracted by the addition of 200 μl CHCl_3_/MeOH 1:1 and vortexing. Phases were separated by centrifugation for 2 min at 9300 g, the lower organic phase was transferred to a new tube and the lipids dried under a low heat vacuum in a Speed-Vac (Univapo 150H). Lipids were re-dissolved in CHCl_3_/MeOH 4:1 and loaded onto a potassium oxalate-pretreated silica gel 60 W F254S TLC plates (Merck) for separation by thin layer chromatography (TLC) in a horizontal tank using CHCl_3_/acetone/MeOH/acetic acid/H_2_O (80/30/26/24/14 v/v) as mobile phase. Dried plates were exposed to Fuij Super RX x-ray films or Kodak Storage Phosphor Screens to quantified by phospho-imaging.

L-α-phosphatidylcholine from chicken egg (PC), sphingomyelin from porcine brain (SM) and L-α-phosphatidylinositol(4,5)bisphosphate from porcine brain, triammonium salt (PI*P*_2_) were purchased from Avanti Polar Lipids, while L-α-phosphatidylserine (PS) from bovine brain and L-α-phosphatidylethanolamine from egg yolk, type III (PE) was obtained from Sigma-Aldrich.

## Mass spectrometry

The PI3Kγ protein band was excised from the gel, reduced with 10 mM DTT, alkylated with 55 mM iodoacetamide and cleaved with porcine trypsin (Promega, Madison, USA) in 50 mM ammonium bicarbonate (pH 8.0) at 37°C overnight [19]. The extracted peptides were analyzed by capillary liquid chromatography tandem mass spectrometry (LC-MSMS) using a Magic C18 100 µm x 10 cm HPLC column (SwissBioanalytics, Switzerland) connected on line to a 4000 Q Trap (MDS Sciex, Concord, Ontario, Canada) as described earlier [20]. Data analysis was done with Mascot [21] searching the protein sequence database UNIPROT_15.6. from Feb 2010 (9421896 sequences; 1248797668 residues). Relative quantification of the peptides was done with multiple reaction monitoring (MRM) using the same instruments and identical chromatographic conditions. The approach was described earlier [22]. The Ser582 site in YE**S^P^**[**582**]LKHPK corresponds well to consensus substrate motifs for PKCβ as determined by peptide library screens [23].

## Protein expression and purification for deuterium exchange studies

The PI3Kγ alone and PI3Kγ/p84 complex were expressed in Sf9 cells using the pFastBac system (Invitrogen). Cells were resuspended in sonication buffer (50 mM Tris/HCl pH 8, 100 mM NaCl, 1 mM PEFA, 25 mM imidazole) and lysed by sonication on ice for 10 minutes. The lysates were ultracentrifuged at 35’000 rpm for 45 minutes at 4 °C in a Ti45 rotor. The soluble cell lysate was filtered through a 0.45 µM filter. Subsequently, the lysate was passed over a Ni-NTA 5 ml Fast Flow column (GE Healthcare) and eluted with an imidazole concentration gradient from 25 to 200 mM. The protein was further purified by ion-exchange HiTrap Q (5ml), Heparin agarose (5ml) and Superdex 200 (16/60) gel-filtration chromatography. The protein was eluted from the gel filtration column in 20 mM Tris pH 7.5, 100 mM NaCl, 5 mM DTT and 1 mM (NH_4_)_2_SO_4_.

## Deuterium exchange sample preparation

5 µl of stock protein solutions (Hs_PI3Kγ-His_6_: 30 µM; Hs_PI3Kγ-His_6_/Mm_p84-His_6_: 35μM) were prepared in 20 mM Tris pH 7.5, 100 mM NaCl, 5 mM DTT and 1 mM ammonium sulfate. Exchange reactions were initiated by addition of 25 µl of a 98% D_2_O solution containing 10 mM HEPES pH 7.2, 50 mM NaCl, and 2 mM DTT, giving a final concentration of 82% D_2_O. Deuterium exchange reactions were allowed to carry on for seven time periods, 3, 10, 30, 100, 300, 1000 and 3000 seconds at 23 ºC. On-exchange was stopped by the addition of 40 µl of quench buffer containing 1.2% formic acid and 0.833 M guanidine-HCl, which lowered the pH to 2.6. Samples were then immediately frozen in liquid nitrogen until mass analysis.

## Protein digestion and peptide identification for DXMS analysis

Different digestion conditions were employed to optimize the peptide digestion map. These optimizations included changing denaturant concentration, flow rate over pepsin, and denaturation time. Peptide identification was performed by running tandem MS/MS experiments using a Sciex QStar Pulsar hybrid QqTOF (Applied Biosystems). Data was analyzed using Mascot software v. 2.2 (Matrix Science) to identify all peptides based on fragmentation and peptide mass, and these identifications were then manually validated using DXMS software (Sierra Analytics) to test for correct m/z state, and the presence of overlapping peptides.

## Measurement of deuterium incorporation

Samples were thawed rapidly on ice and then injected onto an online HPLC system that was immersed in ice. The protein was run over an immobilized pepsin column (Applied Biosystems, Poroszyme®, 2-3131-00) at 50 µl/min, and collected over a 1.7 µm particle C18 peptide trap (2.1 mm x 8 mm, Waters Van-guard) for five minutes. The trap was then switched in line with a 1.7 µm particle, 1 mm x100 mm C18 column (Waters Acquity UPLC) with a vanguard pre-column and peptides were eluted by a 5-45% gradient of buffer A (0.1% formic acid) and buffer B (80% acetonitrile, 20% H_2_O, 0.02% formic acid) over 22 minutes and injected onto a Sciex QStar Pulsar hybrid QqTOF (Applied Biosystems) which collected mass spectra from a range of 350 to 1300 m/z.

Mass analysis of the peptide centroids was performed as described previously using the software DXMS (Sierra Analytics) [24,25]. Briefly, all selected peptides passed the quality control threshold of the software, and were then manually examined for accurate identification and deuterium incorporation. Results are shown as relative levels of deuteration with no correction for back exchange as described previously [26]. The real level of deuteration will be ~25-35% higher than what is shown, based on tests performed with fully deuterated standard peptides. All experiments were repeated in duplicate, and we found that the average error was less than 0.2 Da, and for this reason we consider all changes between conditions greater than 0.5 Da that occur at more than one time point as significant. The resulting deuterium incorporation was graphed versus the on-exchange time. All deuterium exchange data for all peptides analyzed (~202 peptides) is shown in tabular form (Table S1).

## SUPPLEMENTAL REFERENCES

1. Hirsch E, Katanaev VL, Garlanda C, Azzolino O, Pirola L et al. (2000) Central role for G protein-coupled phosphoinositide 3-kinase gamma in inflammation. Science 287: 1049-1053.

2. Leitges M, Plomann M, Standaert ML, Bandyopadhyay G, Sajan MP et al. (2002) Knockout of PKC alpha enhances insulin signaling through PI3K. Mol Endocrinol 16: 847-858.

3. Braz JC, Gregory K, Pathak A, Zhao W, Sahin B et al. (2004) PKC-alpha regulates cardiac contractility and propensity toward heart failure. Nat Med 10: 248-254.

4. Leitges M, Schmedt C, Guinamard R, Davoust J, Schaal S et al. (1996) Immunodeficiency in protein kinase cbeta-deficient mice. Science 273: 788-791.

5. Abeliovich A, Paylor R, Chen C, Kim JJ, Wehner JM et al. (1993) PKC gamma mutant mice exhibit mild deficits in spatial and contextual learning. Cell 75: 1263-1271.

6. Laffargue M, Calvez R, Finan P, Trifilieff A, Barbier M et al. (2002) Phosphoinositide 3-kinase gamma is an essential amplifier of mast cell function. Immunity 16: 441-451.

7. Bondeva T, Pirola L, Bulgarelli-Leva G, Rubio I, Wetzker R et al. (1998) Bifurcation of lipid and protein kinase signals of PI3Kgamma to the protein kinases PKB and MAPK. Science 282: 293-296.

8. Ho SN, Hunt HD, Horton RM, Pullen JK, Pease LR (1989) Site-directed mutagenesis by overlap extension using the polymerase chain reaction. Gene 77: 51-59.

9. Stoyanov B, Volinia S, Hanck T, Rubio I, Loubtchenkov M et al. (1995) Cloning and characterization of a G protein-activated human phosphoinositide-3 kinase. Science 269: 690-693.

10. Pacold ME, Suire S, Perisic O, Lara-Gonzalez S, Davis CT et al. (2000) Crystal structure and functional analysis of Ras binding to its effector phosphoinositide 3-kinase gamma. Cell 103: 931-943.

11. Soltoff SP (2007) Rottlerin: an inappropriate and ineffective inhibitor of PKCdelta. Trends Pharmacol Sci 28: 453-458.

12. Bohnacker T, Marone R, Collmann E, Calvez R, Hirsch E et al. (2009) PI3Kgamma adaptor subunits define coupling to degranulation and cell motility by distinct PtdIns(3,4,5)P3 pools in mast cells. Sci Signal 2: ra27.

13. Schwartz LB, Austen KF, Wasserman SI (1979) Immunologic release of beta-hexosaminidase and beta-glucuronidase from purified rat serosal mast cells. J Immunol 123: 1445-1450.

14. Ozawa K, Szallasi Z, Kazanietz MG, Blumberg PM, Mischak H et al. (1993) Ca(2+)-dependent and Ca(2+)-independent isozymes of protein kinase C mediate exocytosis in antigen-stimulated rat basophilic RBL-2H3 cells. Reconstitution of secretory responses with Ca2+ and purified isozymes in washed permeabilized cells. J Biol Chem 268: 1749-1756.

15. Felmy F, Neher E, Schneggenburger R (2003) Probing the intracellular calcium sensitivity of transmitter release during synaptic facilitation. Neuron 37: 801-811.

16. Ismailov I, Kalikulov D, Inoue T, Friedlander MJ (2004) The kinetic profile of intracellular calcium predicts long-term potentiation and long-term depression. J Neurosci 24: 9847-9861.

17. Grynkiewicz G, Poenie M, Tsien RY (1985) A New Generation of Ca-2+ Indicators with Greatly Improved Fluorescence Properties. J Biol Chem 260: 3440-3450.

18. Wymann MP, Bulgarelli-Leva G, Zvelebil MJ, Pirola L, Vanhaesebroeck B et al. (1996) Wortmannin inactivates phosphoinositide 3-kinase by covalent modification of Lys-802, a residue involved in the phosphate transfer reaction. Mol Cell Biol 16: 1722-1733.

19. Shevchenko A, Wilm M, Vorm O, Mann M (1996) Mass spectrometric sequencing of proteins silver-stained polyacrylamide gels. Anal Chem 68: 850-858.

20. Vichalkovski A, Gresko E, Hess D, Restuccia DF, Hemmings BA (2010) PKB/AKT phosphorylation of the transcription factor Twist-1 at Ser42 inhibits p53 activity in response to DNA damage. Oncogene 29: 3554-3565.

21. Perkins DN, Pappin DJ, Creasy DM, Cottrell JS (1999) Probability-based protein identification by searching sequence databases using mass spectrometry data. Electrophoresis 20: 3551-3567.

22. Hess D, Keusch JJ, Oberstein SA, Hennekam RC, Hofsteenge J (2008) Peters Plus syndrome is a new congenital disorder of glycosylation and involves defective Omicron-glycosylation of thrombospondin type 1 repeats. J Biol Chem 283: 7354-7360.

23. Nishikawa K, Toker A, Johannes FJ, Songyang Z, Cantley LC (1997) Determination of the specific substrate sequence motifs of protein kinase C isozymes. J Biol Chem 272: 952-960.

24. Burke JE, Karbarz MJ, Deems RA, Li S, Woods VLJ et al. (2008) Interaction of group IA phospholipase A2 with metal ions and phospholipid vesicles probed with deuterium exchange mass spectrometry. Biochemistry 47: 6451-6459.

25. Burke JE, Vadas O, Berndt A, Finegan T, Perisic O et al. (2011) Dynamics of the phosphoinositide 3-kinase p110delta interaction with p85alpha and membranes reveals aspects of regulation distinct from p110alpha. Structure 19: 1127-1137.

26. Iacob RE, Pene-Dumitrescu T, Zhang J, Gray NS, Smithgall TE et al. (2009) Conformational disturbance in Abl kinase upon mutation and deregulation. Proc Natl Acad Sci U S A 106: 1386-1391.
